# Hypoxia inducible factors regulate hepatitis B virus replication by activating the basal core promoter

**DOI:** 10.1016/j.jhep.2020.12.034

**Published:** 2021-07

**Authors:** Peter A.C. Wing, Peter Jianrui Liu, James M. Harris, Andrea Magri, Thomas Michler, Xiaodong Zhuang, Helene Borrmann, Rosalba Minisini, Nicholas R. Frampton, Jochen M. Wettengel, Laurent Mailly, Valentina D’Arienzo, Tobias Riedl, Luis Nobre, Michael P. Weekes, Mario Pirisi, Mathias Heikenwalder, Thomas F. Baumert, Ester M. Hammond, David R. Mole, Ulrike Protzer, Peter Balfe, Jane A. McKeating

**Affiliations:** 1 Nuffield Department of Medicine, University of Oxford, Oxford, UK; 2 Chinese Academy of Medical Sciences (CAMS) Oxford Institute (COI), University of Oxford, Oxford, UK; 3 Institute of Virology, Technische Universität München/Helmholtz Zentrum München, Munich, Germany; 4 German Center for Infection Research (DZIF), Munich partner site, Munich, Germany; 5 Department of Translational Medicine, Università del Piemonte Orientale, Novara, Italy; 6 Institute of Inflammation and Immunotherapy, University of Birmingham, Birmingham, UK; 7 Université de Strasbourg, Strasbourg, France; 8 INSERM, U1110, Institut de Recherche sur les Maladies Virales et Hépatiques, Strasbourg, France; 9 Division of Chronic Inflammation and Cancer, German Cancer Research Center (DKFZ), 69120, Heidelberg, Germany; 10 Cambridge Institute for Medical Research, University of Cambridge, Cambridge, UK; 11 Oxford Institute for Radiation Oncology, Department of Oncology, University of Oxford, Oxford, UK

**Keywords:** Hepatitis B, hypoxia, HIF, transcription

## Abstract

**Background & Aims:**

Hypoxia inducible factors (HIFs) are a hallmark of inflammation and are key regulators of hepatic immunity and metabolism, yet their role in HBV replication is poorly defined. HBV replicates in hepatocytes within the liver, a naturally hypoxic organ, however most studies of viral replication are performed under conditions of atmospheric oxygen, where HIFs are inactive. We therefore investigated the role of HIFs in regulating HBV replication.

**Methods:**

Using cell culture, animal models, human tissue and pharmacological agents inhibiting the HIF-prolyl hydroxylases, we investigated the impact of hypoxia on the HBV life cycle.

**Results:**

Culturing liver cell-based model systems under low oxygen uncovered a new role for HIFs in binding HBV DNA and activating the basal core promoter, leading to increased pre-genomic RNA and *de novo* HBV particle secretion. The presence of hypoxia responsive elements among all primate members of the *hepadnaviridae* highlights an evolutionary conserved role for HIFs in regulating this virus family.

**Conclusions:**

Identifying a role for this conserved oxygen sensor in regulating HBV transcription suggests that this virus has evolved to exploit the HIF signaling pathway to persist in the low oxygen environment of the liver. Our studies show the importance of considering oxygen availability when studying HBV-host interactions and provide innovative routes to better understand and target chronic HBV infection.

**Lay summary:**

Viral replication in host cells is defined by the cellular microenvironment and one key factor is local oxygen tension. Hepatitis B virus (HBV) replicates in the liver, a naturally hypoxic organ. Hypoxia inducible factors (HIFs) are the major sensors of low oxygen; herein, we identify a new role for these factors in regulating HBV replication, revealing new therapeutic targets.

## Highlights

• Primate *hepadnaviridae* encode conserved hypoxia response elements.
• Hypoxia inducible factors bind HBV DNA and activate the basal core promoter.
• Pharmacological stabilization of hypoxia inducible factors and low oxygen increases HBV transcription and particle genesis.
• Knockdown studies show a role for both HIF-1α and HIF-2α in regulating HBV transcription.

## Graphical abstract

**Figure:**
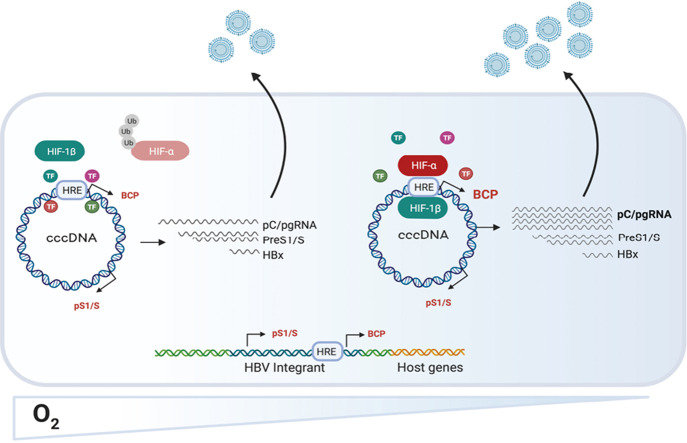

See Editorial, pages 16–18

## Introduction

HBV is a global health problem, with more than 250 million people chronically infected and at least 880,000 deaths/year from HBV-related liver diseases such as cirrhosis and hepatocellular carcinoma (HCC) (WHO, Global hepatitis Report 2017). The HBV vaccine has no effect on chronic infection and current treatments suppress viral replication but are not curative. Chronic infection is associated with a blunted host innate immune response, high viral antigen expression and exhausted antiviral T cell responses that fail to control HBV replication.^1^ In most cases, treatment may be life-long and patients with a functional cure may still develop liver cancer.^2^ Effective antiviral drugs have revolutionized treatments for hepatitis C virus and there is a growing impetus to identify curative therapies for HBV.^3^

HBV is the prototype member of the *hepadnaviridae* family that replicate via episomal copies of a covalently closed circular DNA (cccDNA) genome.^4^ cccDNA is frequently referred to as a viral mini-chromosome, where gene expression is regulated by DNA methylation, host RNA Polymerase II and transcription factors (reviewed in^5^). Viral replication is primarily determined by the size of the cccDNA pool and its transcriptional activity, and host factors such as hepatocyte nuclear factor 4 alpha (HNF4α)^6^ have been reported to regulate HBV replication (reviewed in^7^). The viral genome has 4 promoters (the basal core promoter [BCP], Sp1, Sp2 and Xp) that transcribe 6 major viral RNAs of decreasing length with a common 3’ polyadenylation signal. These RNAs include: pre-core (pC) that encodes the e antigen (HBeAg); pre-genomic (pgRNA) that is translated to yield core protein (HBcAg) and polymerase; preS1, preS2 and S RNAs encoding the surface envelope glycoproteins and the X transcript for the multi-functional x protein (HBx). Encapsidated pgRNA is reverse-transcribed by the viral polymerase to generate new DNA genomes that can be reimported to the nucleus to maintain the cccDNA pool or are enveloped and secreted as infectious particles.^4^ Defining the role of host factors that regulate HBV pgRNA genesis and half-life will increase our understanding of the HBV life cycle and enable the design of more effective antiviral approaches.

Viral gene expression is shaped by the cellular microenvironment and one important factor to consider is local oxygen tension. The liver receives oxygenated blood from the hepatic artery and oxygen-depleted blood via the hepatic portal vein, resulting in an oxygen gradient of 8–4% between the periportal and pericentral areas, respectively.^8^ This oxygen gradient associates with liver zonation, a phenomenon where hepatocytes show distinct functional and structural heterogeneity across the liver.^9^ Mammalian cells adapt to low oxygen through an orchestrated transcriptional response regulated by hypoxia inducible factor (HIF). This transcription factor, comprising HIF-1β and HIF-1α or HIF-2α subunits, is regulated by oxygen-dependent and independent stress signals such as inflammation and oxidative stress.^10^^,^^11^ The heterodimeric HIF complex binds a consensus RCGTG(C) motif or hypoxic responsive element (HRE) in the promoter and enhancer regions of responsive genes. When oxygen is abundant, newly synthesised HIFα subunits are hydroxylated by prolyl-hydroxylase domain (PHD) proteins and rapidly targeted for proteasomal degradation. In contrast, when oxygen is limited, these HIFα subunits translocate to the nucleus, dimerize with HIF-1β and regulate the transcription of host genes involved in cell metabolism and immune regulation.^12^ The majority of reports studying HBV replication *in vitro* are performed at atmospheric oxygen levels (18%) where HIFs are inactive, so their role in viral replication has been overlooked. We evaluated the effect of HIF signaling on HBV replication and uncovered a positive role for HIFs in activating viral transcription that could inform future therapeutic strategies.

## Materials and methods

### HBV, cells and hypoxic culture

HBV (Genotype D) was purified from HepAD38 cells and the infectious titer measured as previously reported.^13^ HepG2 cells expressing sodium taurocholate co-transporting polypeptide (NTCP) (Stefan Urban, Heidelberg University) were cultured in DMEM with 100 U/ml penicillin/0.1 mg/ml streptomycin/10% FCS/glutamax/non-essential amino acids. HepG2-NTCP cells were seeded on collagen coated plasticware and infected with HBV (multiplicity of infection 200–400 genome equivalents/cell) with 4% polyethylene glycol 8000 for 6 h. Viral inoculum was removed, cells washed 3x with PBS and infected cells maintained in DMEM-10% FCS in the presence or absence of 2.5% DMSO. For hypoxic cultures, cells were incubated in a Galaxy 48R incubator (Eppendorf) with variable oxygen tension; unless otherwise stated all incubations were for 72 h. Normoxic cells were cultured at 5% CO_2_ and 18% oxygen.

For further details regarding the materials and methods used, please refer to the CTAT table and supplementary information.

## Results

### Hypoxia inducible factors activate the HBV basal core promoter

**Fig. 1:**
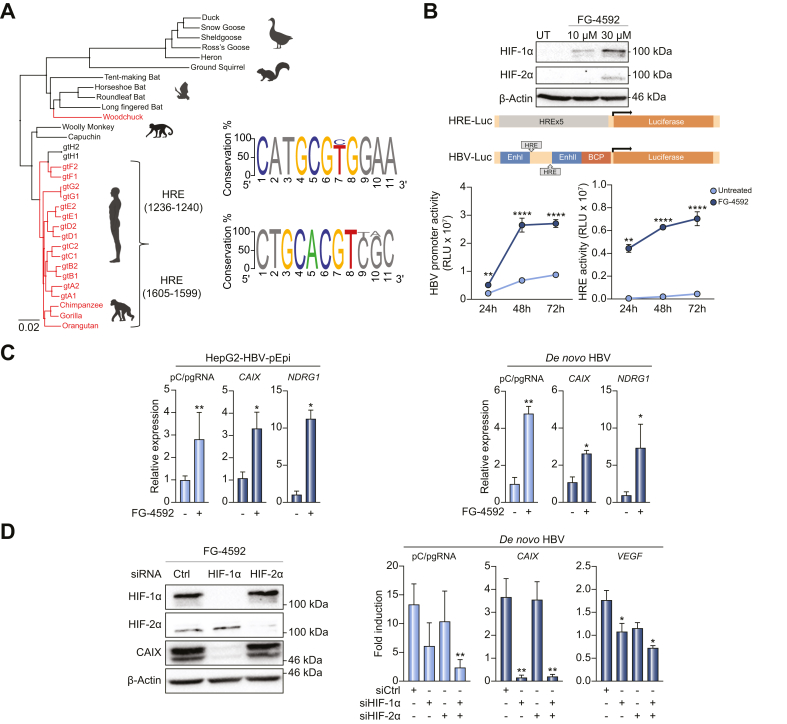
HIFs positively regulate HBV transcription. (A) Conservation of HREs amongst diverse HBV lineages. Conservation of hypoxia responsive elements at positions 1236–1240 and 1605–1599 were assessed amongst 7,313 HBV sequences^14^ and consensus plots generated. NCBI referent full-length *hepadnaviridae* genome sequences were aligned and a Neighbor Joining tree constructed, where branches highlighted in red denote HRE containing genomes. (B) HIFs induce HBV promoter activity. HepG2-NTCP cells expressing an HRE or HBV EnhI/II and BCP construct driving luciferase (HBV-Luc) were treated with PHD inhibitor FG-4592 (30 μM) and promoter activity assessed at 24 h intervals for 72 h. HIF-1α and HIF-2α expression was confirmed by western blot. Statistical analysis was performed using a 2-Way ANOVA (∗∗*p ≤*0.01, ∗∗∗∗*p ≤*0.0001). (C) HIFs promote transcription in HBV *de novo* infection. HepG2 cells supporting episomal copies of HBV DNA (HepG2-HBV-pEpi) were treated with FG-4592 (30μM) for 72 h and pC/pgRNA, host genes *CAIX* and *NDRG1*, were quantified by qPCR. HBV-infected (MOI 200) HepG2-NTCP cells were treated with FG-4592 (30 μM) and pC/pgRNA, *CAIX* and *NDRG1* expression was quantified 72 h post infection. (D) HIF KD studies. siRNAs targeting HIF-1α or HIF-2α were delivered into HepG2-NTCP cells that were infected with HBV (MOI 200) and treated with FG-4592 (30 μM) for 72 h. siRNA knockdown was confirmed by qPCR of host genes, CAIX (HIF-1α dependent) and VEGFA (HIF-1α and -2α co-dependent) and cell lysates probed for HIF-1α, HIF-2α and CAIX expression. HBV pC/pgRNA levels quantified by PCR. Data is presented as fold induction relative to the 18% oxygen control for each condition. Statistical analysis was performed using a one-way ANOVA (∗*p ≤*0.05, ∗∗*p ≤*0.01). BCP, basal core promoter; HIFs, hypoxia inducible factors; HRE, hypoxic responsive element; KD, knockdown; MOI, multiplicity of infection. (This figure appears in color on the web.)

To investigate a role for HIFs in the HBV life cycle, we first assessed whether the viral genome encodes HREs. Screening >7,000 HBV sequences available in the HBV database^14^ identified HREs within Enhancer I (1236–1240) and an antisense motif between Enhancer I and II (EnhI/II) (1604–1599) (Fig. 1A). It is interesting to note that both HREs are present in HBV sequences obtained from Bronze age and medieval samples.^15^ Analysis of *hepadnaviridae* NCBI referent genomes^16^ shows that, with the exception of HBV genotype H, all human, higher primate, and woodchuck viruses encode both motifs (Fig. 1A). Monkey, bat, rodent and avian viral genomes lack the motifs, suggesting a conserved evolutionary role for HIFs in the regulation of primate members of this family. To evaluate this hypothesis we stabilized HIF expression using the licensed PHD inhibitor FG-4592 (Roxadustat).^17^ Since HIFα isoform expression can vary between cell types we show that FG-4592 induced HIF-1α and HIF-2α expression in HepG2 cells derived from a human HCC (Fig. 1B). Transfecting HepG2 cells with plasmids encoding EnhI/II and the basal core promoter (HBV-Luc) or control HRE-luciferase showed that FG-4592 induced a significant, time-dependent activation of both reporters (Fig. 1B).

To extend these observations we used 2 model systems to investigate a role for HIFs in regulating viral transcriptional activity: HepG2 cells with an episomal replicating HBV (HepG2-pEpi-HBV)^18^ and HepG2-NTCP cells that support *de novo* HBV infection. The BCP drives transcription of pC and pgRNA from 2 start sites that are only 70 base pairs apart^19^ and since our PCR cannot discriminate between these viral-encoded RNAs, we label transcripts as pC/pgRNA to represent the sum of both RNAs. FG-4592 induced a significant increase in pC/pgRNA and HIF-regulated genes carbonic anhydrase IX (CAIX) and N-myc downstream regulated 1 (NDRG1) in both models (Fig. 1C). Since HIF-1α and HIF-2α can show non-overlapping and opposing functions^11^ we were interested to investigate their individual roles in regulating viral transcription. We transiently silenced HIF-1α or HIF-2α, individually or together, in HepG2-NTCP cells prior to infecting with HBV in the presence or absence of FG-4592. We demonstrate effective silencing by western blotting for HIF expression and by quantifying *CAIX* or vascular endothelial growth factor (*VEGF*) mRNA levels (HIF-1α and HIF-1α/HIF-2α regulated host genes, respectively) (Fig. 1D). The FG-4592-dependent increase in pC/pgRNA in the infected cells was ablated by the combined HIF-1α and HIF-2α silencing, demonstrating a role for both HIFα isoforms in regulating promoter activity.

**Fig. 2:**
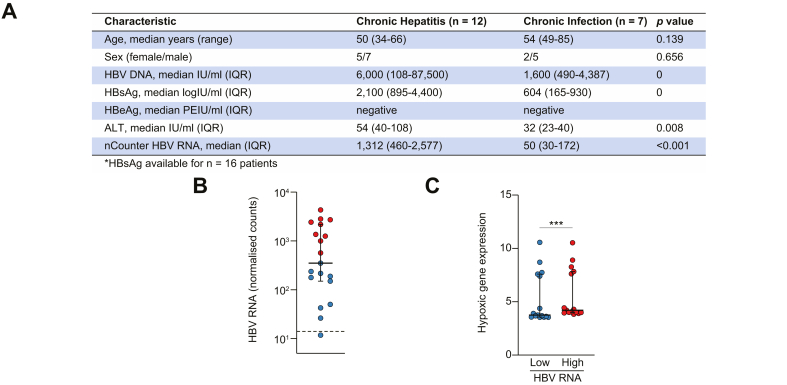
Hypoxic gene expression associates with HBV RNA levels in chronic infection. (A) Clinical characteristics of CHB cohort. (B) HBV RNA in the CHB biopsies was quantified by nCounter and classified into high (Normalized count >350: n = 9 and red symbols) or low (Normalized count <350: n = 10 and blue symbols) based on their deviation from the median, where the dashed line indicates the assay cut-off defined by analyzing liver RNA samples from uninfected control patients (n = 8). (C) HBV RNA positively associates with the expression of 15 HIF-regulated genes (*BNIP3, BNIP3L, CA9, EPO, FAM115C, FGF11, GLUT1, IGLON5, LCN15, LOX, NDRG1, PFKFB4, SPAG4, TNS1, VEGFA*). Statistical testing was performed using Wilcoxon match-pairs signed rank test (*p* = 0.00012). CHB, chronic hepatitis B; HIF, hypoxia inducible factor. (This figure appears in color on the web.)

As our cell-based studies predict an association between HIF-transcriptional activity and viral RNAs in the infected liver, we quantified hypoxic gene transcripts and HBV RNA in liver biopsies from patients diagnosed with chronic hepatitis (n = 12) or chronic infection (n = 7) following the 2017 EASL guidelines^20^ (Fig. 2A). We recently reported increased expression of hypoxia regulated gene transcripts in chronic hepatitis B (CHB),^21^ enabling us to identify hepatic HIF-target genes for inclusion in a customized nCounter (Nanostring) array together with a probe to measure HBV RNA. The HBV probe detected a 400-fold range in viral RNA counts amongst the CHB samples (Fig. 2B), enabling us to stratify patients into 2 groups (nCounter score low <350 and high >350). We found a significant positive association between HIF-gene expression and HBV RNA (Fig. 2C), consistent with our *in vitro* studies suggesting a role for HIFs in positively regulating HBV transcription.

### HIFs regulate viral transcripts in HBV transgenic mice.

**Fig. 3:**
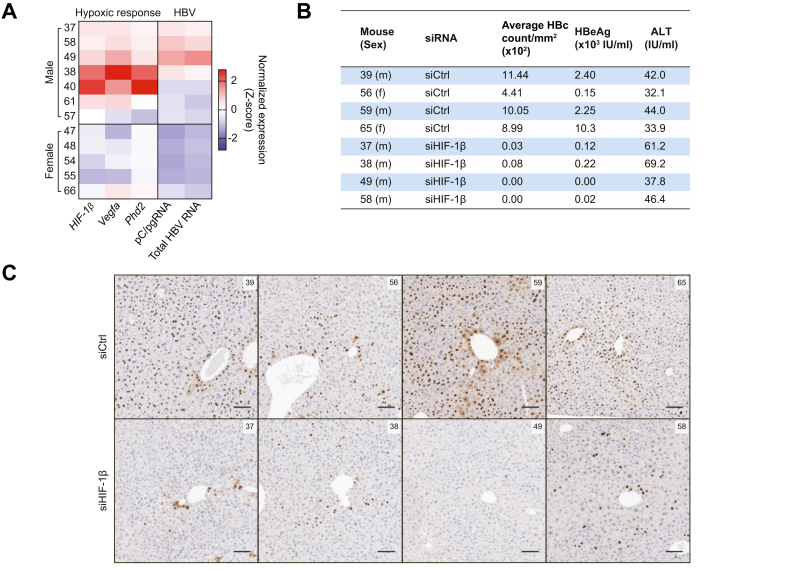
HIFs regulate viral transcription in HBV transgenic mice. (A) siRNAs targeting HIF-1β were delivered into male and female HBV1.3 transgenic mice and animals were culled after 1 week. RNA was isolated from liver biopsies and transcript levels of *HIF-1β*, *Vegfa* and *Phd2*, together with pC/pgRNA and total viral RNA quantified. The amount of each transcript was normalized (Z-score) and presented as a heat map (suppression = red, enhancement=blue). (B) HBcAg expression in the liver was assessed by measuring the number of antigen expressing cells/mm^2^. Peripheral HBeAg, HBsAg and ALT levels were quantified in siCTRL and HIF-1β silenced mice and tabulated per individual mouse. (C) HBcAg staining from fixed murine liver sections. 20x images are shown with scale bars representing 100 μm. ALT, alanine aminotransferase; HIFs, hypoxia inducible factors; pC/pgRNA, pre-core/pre-genomic RNA. (This figure appears in color on the web.)

HBV can only infect humans and primates and no immune competent animal models are available that support natural HBV infection. The HBV transgenic mouse model (HBVtg)^22^ of chronic infection transcribes viral RNAs from an integrated 1.3 overlength genome and, despite the lack of cccDNA, has been used to study host pathways regulating viral transcription (reviewed in^7^). Since both HIFα subunits dimerize with HIF-1β to regulate gene transcription we transiently silenced HIF-1β by siRNA injection and quantified viral parameters in the liver and periphery. To assess the efficacy of the silencing we measured mRNA levels of HIF-1β, the HIF-regulated host genes VEGF and PHD2, the key oxygen sensor that regulates HIF expression. We observed a coordinated reduction of all 3 gene transcripts in 6 of 7 male mice receiving siRNAs targeting HIF-1β and a negligible effect of the siRNA-HIF-1β in 5 female mice (Fig. 3A, Fig. S1). We noted reduced pC/pgRNA and total viral RNAs in 4 of 6 siRNA-HIF1β treated male mice compared to siControl treated animals (Fig. 3B), demonstrating a role for HIFs in regulating HBV transcription. Alanine aminotransferase values were comparable in all mice suggesting a negligible cytotoxic effect of the siRNA-HIF-1β treatment. Furthermore, visual inspection of H&E-stained liver biopsies from siCtrl or siRNA-HIF-1β treated animals showed no evidence of hepatocyte proliferation. We observed a reduced frequency of HBcAg-expressing hepatocytes, peripheral HBeAg and HBsAg levels in the siRNA-HIF-1β treated male mice (Fig. 3B,C). Core antigen (HBcAg) expression showed a peri-central staining pattern (Fig. 3C), consistent with this area of the liver representing a low oxygen environment.^23^ These data highlight a role for HIFs in regulating HBV transcription in this transgenic mouse model.

### Hypoxia inducible factors bind HBV DNA

**Fig. 4:**
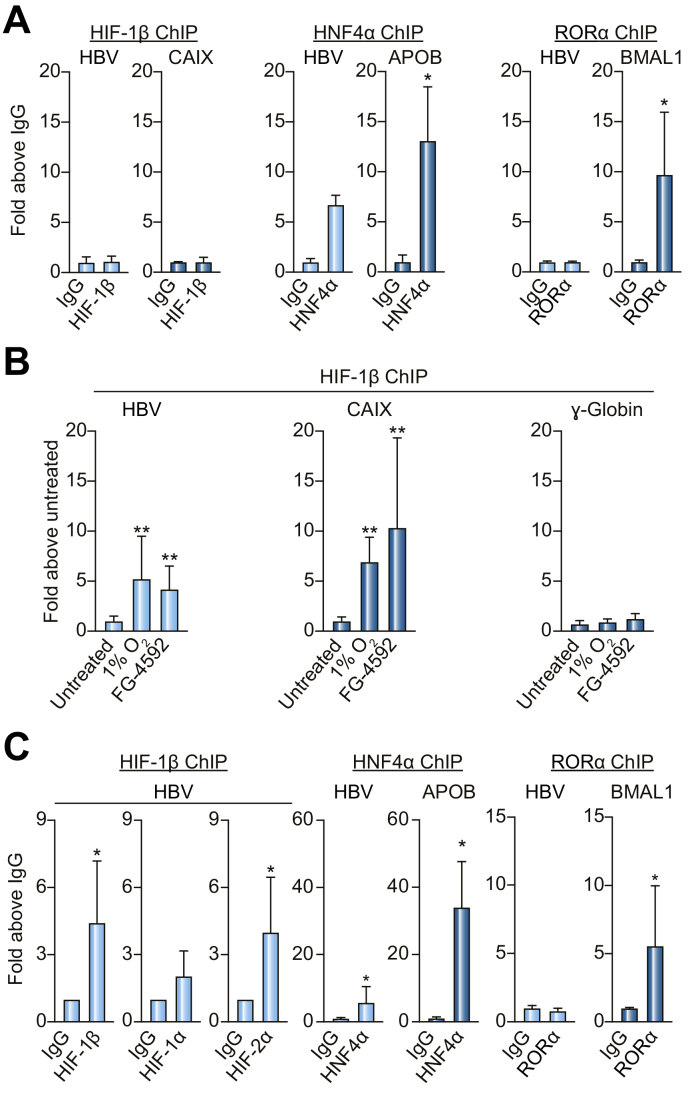
HIFs bind HBV DNA. (A) Limited evidence for HIF-1β binding HBV under normoxic conditions. Chromatin extracts from HepG2-HBV-pEpi cells cultured under 18% O_2_ were immunoprecipitated with anti-HIF-1β, anti-HNF4α, anti-RORα or an irrelevant IgG control. PCR for HBV cccDNA and host genes *CAIX, APOB* and *BMAL1* was performed and data presented relative to % of input of the IgG. Data are the mean + SD of 2 independent experiments and statistical analysis performed with Mann-Whitney *U* test (∗*p ≤*0.05). (B) Hypoxia and FG-4592 potentiates HIF binding to HBV DNA. Chromatin extracts from HepG2-HBV-pEpi cells cultured at 18% or 1% O_2_ or treated with FG-4592 (30 μM) for 24 h were immunoprecipitated with anti-HIF1β or irrelevant IgG control. PCR for HBV cccDNA and HIF-regulated gene CAIX and the non-HIF housekeeper gene γ-Globin was performed and the data presented relative to untreated (18% O_2_) samples. Data are representative of mean + SD of 2 independent experiments and statistical analysis was performed with a Mann-Whitney *U* test (∗*p ≤*0.05). (C) HIFs bind HBV DNA in infected liver chimeric mice. Chromatin from HBV-infected mouse liver tissue was immunoprecipitated with antibodies specific for HIF-1β, HIF-1α, HIF-2α, HNF4α, RORα along with an irrelevant IgG control. Following immunoprecipitation, the DNA was analyzed by qPCR with primers spanning the binding sites of the respective transcription factors on the HBV genome. % of input was calculated and data presented relative to the IgG control and shown as the mean + SD for 2 mice. cccDNA, covalently closed circular DNA; HIFs, hypoxia inducible factors; qPCR, quantitative PCR.

To assess whether HIFs bind HBV DNA we isolated chromatin from HepG2-pEpi-HBV cells and quantified HIF-1β binding to episomal genomes by chromatin immunoprecipitation and quantitative PCR (ChIP-qPCR).^24^ Under normoxic conditions we failed to precipitate HIF-1β with any target gene (Fig. 4A), whereas HNF4α bound viral DNA and the promoter of Apolipoprotein B (APOB)^6^^,^^25^ (Fig. 4A). The retinoic acid receptor-related orphan receptor-α (RORα) is an important regulator of circadian rhythm and hepatic lipid metabolism^26^ and, to the best of our knowledge, does not interact with the HBV genome. As a control for these ChIP experiments, we showed that RORα bound its circadian target gene BMAL1 but failed to bind HBV DNA (Fig. 4A). *In vitro* studies to stabilize HIFs are routinely performed under 1% oxygen^27^ and culturing cells under these conditions or treatment with FG-4592 induced HIF-1β binding to HBV DNA and the host target gene CAIX (Fig. 4B). HIF-1β showed no binding to the γ-Globin promoter, a known housekeeping gene lacking an HRE motif (Fig. 4B). To assess whether HIFs bind HBV DNA *in vivo* we studied infected chimeric human liver FNRG mice.^28^ HBV-infected mice were sacrificed at 4 weeks post-infection and viral replication was confirmed by measuring hepatic cccDNA along with peripheral HBV DNA, HBeAg and HBsAg (Fig. 4C). ChIP-qPCR using primers spanning the BCP^24^ showed enriched HIF-1β, HIF-1α and HIF-2α binding to viral genomes relative to an irrelevant IgG (Fig. 4C). In agreement with our earlier *in vitro* experiments HNF4α also bound viral DNA whereas there was no evidence for an interaction with RORα (Fig. 4C). In summary, these experiments provide clear evidence for HIF-1β binding HBV DNA following FG-4592 or low oxygen treatments. Furthermore, HIF-1α and HIF-2α complexed with HBV DNA in infected human hepatocytes isolated from liver chimeric mice, without any specific treatments to stabilize HIFs, showing the presence of these complexes *in vivo*.

### A low oxygen environment activates HBV BCP and associated transcripts

**Fig. 5:**
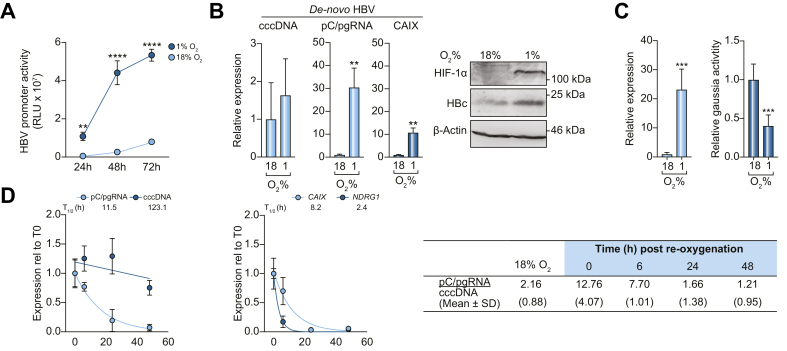
Low oxygen promotes HBV transcription. (A) Low oxygen activates HBV BCP. HepG2-NTCP cells expressing HBV EnhI/II and BCP construct driving luciferase were cultured at 18% or 1% oxygen. Viral promoter activity was assessed by measuring luciferase activity after 24, 48 and 72 h. Statistical analysis was performed using a 2-way ANOVA (∗∗*p ≤*0.01, ∗∗∗∗*p ≤*0.0001). (B) Low oxygen promotes *de novo* HBV infection associated transcription. HepG2-NTCP cells were infected with HBV at an MOI of 200 and incubated at 18% or 1% oxygen for 72 h. cccDNA, pC/pgRNA and *CAIX* mRNA levels were quantified by qPCR and HBcAg assessed by western blotting. (C) HepG2-NTCP cells were infected with a modified HBV encoding Gaussia luciferase and incubated under 18% or 1% oxygen for 72 h. pC/pgRNA levels were quantified and Gaussia luciferase activity measured. (D) The dynamic nature of low oxygen-regulation of HBV pC/pgRNA. HBV-infected HepG2-NTCP cells cultured at 1% oxygen for 72 h were perfused with 18% oxygen and samples collected at the indicated times. HBV cccDNA and pC/pgRNA along with host genes *CAIX* and *NDRG1* were quantified and used to estimate mRNA half-life. cccDNA transcriptional activity at 1% oxygen and during the period of re-oxygenation is shown. All data presented as the mean ± SD derived from at least 3 independent experiments consisting of 3 replicates per condition. Statistical analysis was performed using a Mann-Whitney *U* test (∗*p ≤*0.05, ∗∗*p ≤*0.01, ∗∗∗∗*p ≤*0.0001). cccDNA, covalently closed circular DNA; HIFs, hypoxia inducible factors; pC/pgRNA, pre-core/pre-genomic RNA; qPCR, quantitative PCR.

To investigate the functional consequences of a low oxygen environment on HBV transcription activity we cultured HepG2-NTCP cells under 1% oxygen and measured promoter activity and pC/pgRNA levels in *de novo* infected cells. Under low oxygen conditions HepG2-NTCP cells express both HIFα isoforms and show a time-dependent increase in promoter activity (Fig. 5A), increased pC/pgRNA levels and HBcAg expression (Fig. 5B). We observed a significant increase in the level of secreted HBV DNA under low oxygen conditions using 2 independent model systems (Ad-HBV transduced HepG2-NTCP and HepG2.2.15 cells) (Fig. S2), consistent with reports showing that a modest increase in HBcAg promotes cytoplasmic HBV replication.^29^ Low oxygen had no impact on cccDNA levels, consistent with our interpretation that HIFs positively regulate BCP activity. To further explore this conclusion, we infected hypoxic HepG2-NTCP cells with a transcriptionally active but replication deficient HBV reporter virus (rHBV-Gluc) and showed a low oxygen-dependent increase in pC/pgRNA (Fig. 5C). This virus encodes Gaussia luciferase under the control of an exogenous transthyretin promoter within the S open reading frame.^30^ We observed a modest reduction in reporter activity in the hypoxic infected cells, demonstrating the promoter-dependency of low oxygen in regulating HBV transcription. HIFs are rapidly degraded under normoxic conditions to facilitate dynamic cellular transcriptional responses to fluctuating oxygen levels. We were interested to assess the impact of re-oxygenation on HBV transcription. HBV-infected HepG2-NTCP cells were maintained in 1% oxygen for 72 h, moved to 18% oxygen and sampled at 0, 6, 24 and 48 h post-oxygenation. We quantified cccDNA and transcript levels of *pC/pgRNA, CAIX* and *NDRG1*. cccDNA levels showed no significant change over the period of re-oxygenation, however, pC/pgRNA levels declined rapidly, with a 50% reduction by 11 h compared to 8 h and 2 h for *CAIX* and *NDRG1* RNAs, respectively (Fig. 5D). These data clearly show that low oxygen regulates cccDNA transcriptional activity, highlighting the dynamic nature of this phenotype.

In the adult liver hepatocytes are non-proliferating and the majority of *in vitro* experiments studying HBV replication use DMSO to arrest cell proliferation. We noted that culturing HepG2-NTCP cells under 1% oxygen arrests cells (Fig. S3), providing a physiological method to limit cell proliferation. DMSO can induce wide-ranging effects on host gene expression^31^ and we evaluated the effect of low oxygen on pC/pgRNA levels in DMSO-arrested HepG2-NTCP cells, comparing a naïve *de novo* infection to a pre-established infection. In both cases we noted a blunted response of low oxygen to regulate pC/pgRNA in the DMSO-arrested cells (Fig. S4A,B). Screening a panel of hypoxia genes by PCR-array showed a reduced activation of both HIF-1α and HIF-2α regulated genes in DMSO-treated HepG2 cells cultured in 1% oxygen (Fig. S5).

Primary human hepatocytes (PHHs) are considered the gold standard for studying HBV replication. However, PHHs can de-differentiate and lose hepatocyte-specific function *in vitro*.^32^ One approach to limit their de-differentiation is to culture them in DMSO-containing media. Since DMSO blunts HepG2 cellular response to hypoxia, we cultured PHHs and HepG2-NTCP cells in 5%, 3% or 1% oxygen for 48 h and showed negligible expression of HIF-1α and greater levels of HIF-2α in PHHs compared to HepG2-NTCP (Fig. S5). We observed a more dominant pattern of HIF-2α regulated genes in PHHs compared to HepG2-NTCP cells (Fig. S5C). To understand whether HIF-2α transcriptional activity dominates in the HBV-infected liver we investigated the relative contribution of HIF-1α or HIF-2α activity in CHB biopsy samples.^21^ 38 of the hypoxia genes showing increased expression in CHB were defined as HIF-1α (n = 26), HIF-2α (n = 11) or co-regulated (n = 1) genes,^33^ showing clear evidence for both HIF-1α and HIF-2α transcriptional activity *in vivo*. These data show the complexities of studying viral and host transcriptional responses to low oxygen in PHHs *ex vivo.*

### Oxygen-regulation of episomal and integrated HBV DNA

**Fig. 6:**
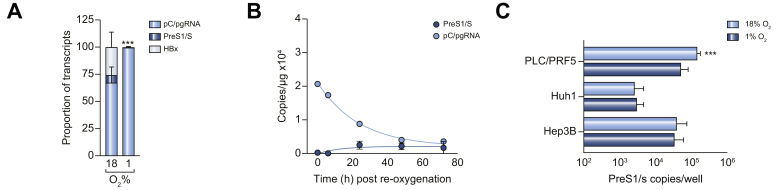
Oxygen-dependent differential regulation of episomal or integrated HBV DNA. (A) Quantification of the major HBV transcripts under low oxygen. Infected HepG2-NTCP were cultured at 18% or 1% oxygen for 72 h. pC/pgRNA, PreS1/S and HBx RNA transcripts were quantified by PCR using specific primers with copy number inferred from a standard curve, adjusted to the host housekeeper gene, β2M. (B) Effect of oxygen reperfusion on HBV preS1/S RNA levels. HBV-infected HepG2-NTCP cells cultured under 1% oxygen for 72 h were perfused with 18% oxygen and samples collected at the indicated times. HBV pgRNA and preS1/S RNA levels were quantified by qPCR. Data presented are the mean ± SD derived from 3 independent experiments consisting of 3 replicates per condition. (C) Hepatoma lines bearing viral integrants were treated as in (A) and preS1/S RNAs quantified and data presented are the mean ± SD derived from 3 independent experiments consisting of 3 replicates per condition. Statistical analysis was performed using a Mann-Whitney *U* test (∗∗∗*p ≤*0.001). pC/pgRNA, pre-core/pre-genomic RNA; qPCR, quantitative PCR.

In the human genome HIFs bind motifs that can regulate distant gene promoters. We previously reported a qPCR technique to quantify the relative abundance of HBV RNAs^34^ and used this method to assess the effect of low oxygen on the pattern of viral RNAs in infected HepG2-NTCP cells. pC/pgRNA was the major viral-encoded RNA in infected HepG2-NTCP cells, consistent with a recent report using 5’RACE to study HBV transcripts.^19^ Low oxygen increased pC/pgRNA levels compared to preS1/S or HBx RNAs in HepG2-NTCP cells (Fig. 6A). Furthermore, following oxygen reperfusion of hypoxic infected cells, preS1/S RNA levels showed no significant change, suggesting a minimal role for low oxygen in regulating their genesis or estimated half-life (Fig. 6B). Recent reports highlight a role for HBV integrants in driving HBsAg expression.^35^^,^^36^ Since integration of the linear viral DNA generated during viral replication may separate the HREs from downstream transcription initiation sites, we hypothesize that integrated copies of HBV will be oxygen-insensitive. We used 3 well-characterized hepatoma lines: PLC/PRF5, Hep3B and Huh-1 that harbor transcriptionally active integrated genomes.^37^^,^^38^ Low oxygen had a minimal effect on preS1/S2 RNA levels (Fig. 6C), with a modest reduction in transcripts in PLC/PRF5 cells, and no significant changes in HBsAg expression. We confirmed the integrant lines were responsive to low oxygen, showing an approximate 10-fold induction in *CAIX* RNA levels. These experiments show the differential effect of HIFs in the regulation of transcription of episomal cccDNA and HBV integrants.

## Discussion

Oxygen sensing is a fundamental cellular process that shapes the liver transcriptome. We identify a role for HIFs in activating the HBV core promoter and increasing pC/pgRNA, HBcAg expression and secreted viral DNA. Our ChIP experiments show HIF binding to HBV DNA in hypoxic infected cells and from HBV-infected human liver chimeric mice *in vivo*, supporting a direct role for HIFs in the regulation of HBV transcription. Furthermore, we translate these observations into CHB and show a positive association between hypoxic gene expression and viral RNAs, consistent with a role for HIFs in regulating HBV *in vivo*. HBV encodes 2 HREs in its compact genome and we show their evolutionary conservation among primate members of the *hepadnaviridae.*

Silencing HIF-1β in male HBVtg mice reduced pC/pgRNA and total RNA levels, the frequency of HBcAg-expressing hepatocytes, and peripheral HBeAg and HBsAg levels, revealing a role for HIFs in regulating viral transcription in this model. Analyzing published single cell-RNA sequences of mouse liver^23^ for transcriptional activators and repressors reported to regulate HBV replication (reviewed in^39^) showed no zonal patterns of expression, consistent with a role for HIFs in regulating viral transcription in this mouse model. We noticed a discrepancy between the male and female mice in our study, where HIF-1β silencing was ineffective in females, consistent with reports showing a greater response in male rats to chronic intermittent hypoxia compared to females.^40^^,^^41^ Furthermore, ovariectomy rendered the 2 sexes equally responsive to hypoxia.^41^ Estrogen signaling was reported to inhibit both HIF-1α transcriptional activity^42^ and HBV transcription,^43^^,^^44^ providing a potential explanation for the sex-dependent differences noted in our model. A recent in-depth proteomic study investigating the molecular basis of sex difference in zebrafish responses to hypoxia preconditioning identified a complex network of signaling pathways.^45^ Hypoxic gene expression and HBV RNA levels in our CHB samples were independent of sex, however, this may reflect the post-menopausal age of females in our small cohort and is worthy of further investigation.

Hypoxia is known to induce a broad range of cellular changes beyond those regulated by HIFs.^46^ Importantly, we observed higher pC/pgRNA in *de novo* HBV-infected cells cultured under low oxygen compared to those treated with the PHD inhibitor FG-4592 (Roxadustat), suggesting a potential role for other members of the oxygenase family in the regulation of HBV replication. To investigate this possibility, we completed a transcriptional and proteomic analysis of hypoxic human hepatoma cells (Fig. S6) and observed negligible changes in the expression of host activators or repressors previously reported to regulate HBV replication. A recent study using chemical mimetics (CoCl_2_ and dimethyloxalylglycine) to stabilize HIFs reported increased DNASE1 levels and a reduction in HBV genome copies,^47^ however, our hypoxic studies showed no impact on DNASE1 expression that may reflect differences in the model systems used.

Integration of the HBV genome is associated with HCC development^48^^,^^49^ and the introduction of HREs could regulate downstream targets such as oncogenes that could be activated in the hypoxic tumor environment. HBV integrants are thought to be the major source of HBsAg expression in chronic disease that have been associated with exhausted antiviral T cell responses.^1^^,^^50^ Our observation that integrant encoded preS1/S transcripts are oxygen-insensitive is relevant if one considers the hypoxic nature of the HCC environment^21^^,^^51^ and how this could influence HBsAg expression, a biomarker for monitoring patients’ response to new therapies.^52^

In summary, we demonstrate a new role for HIFs to positively regulate HBV transcription. A hypoxic environment was also reported to potentiate hepatitis C virus replication via HIF-dependent^53^^,^^54^ and independent^55^ pathways. Hypoxia can have variable effects on virus replication, most likely reflecting the differing oxygen tensions at the sites of replication.^56^ HBV, along with other members of the *hepadnaviridae* family, may have evolved to replicate and exploit the HIF-signaling pathway to persist in the low oxygen environment of the liver.

Our observations raise questions as to how cellular hypoxia translates to humans, both in terms of HBV replication, hepatic immunity and response to new therapies. CHB reflects a dynamic interaction between the virus and host immune system cells, where active hepatitis associates with increased cccDNA transcription.^57^ Our data showing a role for HIFs in promoting HBV transcription provide an explanation for how inflammatory responses may potentiate HBV replication. The recent licensing of HIF-prolyl hydroxylase inhibitors as erythropoiesis-stimulating agents for the treatment of anemic patients with chronic kidney disease in China and Japan^58^ could impact HBV replication and is worthy of further study. Finally, pharmacological agents that inhibit HIF signaling-pathways^10^ may be considered for further exploration in clinical trials. Understanding the biology of HBV under more physiological conditions will reveal novel therapeutic targets and provide an environment for innovative antiviral drug screening.

### Abbreviations

BCP, basal core promoter; CAIX, carbonic anhydrase IX; cccDNA, covalently closed circular DNA; CHB, chronic hepatitis B; ChIP, chromatin immunoprecipitation; HCC, hepatocellular carcinoma; HIFs, hypoxia inducible factors; HRE, hypoxic responsive element; NDRG1, N-myc downstream regulated 1; NTCP, sodium taurocholate co-transporting polypeptide; pC, pre-core; pgRNA, PHD, prolyl-hydroxylase domain; qPCR, quantitative PCR; RORα, retinoic acid receptor-related orphan receptor-α; VEGF, vascular endothelial growth factor.

## Financial support

JAM laboratory is funded by 10.13039/100010269Wellcome Trust IA 200838/Z/16/Z, 10.13039/501100000265MRC project grant MR/R022011/1 and Chinese Academy of Medical Sciences (CAMS) Innovation Fund for Medical Science (CIFMS), China (grant number: 2018-I2M-2-002). UP laboratory is funded by the German research foundation via the collaborative research center TRR179 (project TP14; UP). TM received a Gerok stipend by TRR179. UP and JAM collaboration was supported by funding by the Institute for Advanced Study with the support of the Technical University of Munich via the German Excellence Initiative and EU 7th Framework Program under grant agreement number 291,763. JW received a stipend by the 10.13039/501100001655German Academic Exchange Service (DAAD). MW lab is funded by a Wellcome Trust Senior Clinical Research Fellowship 108070/Z/15/Z. MP was (partially) funded by the Università del Piemonte Orientale, FAR-2017. TFB was supported by ARC, Paris (TheraHCC2 IHUARC201901299), 10.13039/501100003323ANRS, LabEx HepSys and Inserm Plan Cancer. DRM laboratory is funded by the 10.13039/501100000272National Institute for Health Research (NIHR-RP-2016-06-004), the Deanship of Scientific Research, King Abdulaziz University, 10.13039/501100011821Ministry of High Education for Saudi Arabia. JAM, UP, TFB and MH were supported by the 10.13039/501100000780European Union (EU H2020-667273-HEPCAR).

## Authors’ contributions

PW designed experiments, analyzed and curated data and co-wrote the manuscript; PJL designed experiments, analyzed and curated data and edited the manuscript; JMH designed experiments, analyzed and curated data and edited the manuscript; AM conducted experiments, analyzed and curated data; TM conducted experiments; XZ conducted experiments and analyzed data; HB conducted experiments and analyzed data; RM provided clinical samples; NRF conducted experiments; JW provided reagents; LM conducted experiments; VD conducted experiments; TR conducted experiments; LN analyzed data; MPW analyzed data; MP provided clinical material and advice; MH provided reagents and advice; TFB provided reagents and advice; EH provided advice and edited manuscript; DRM provided advice and reagents; UP provided advice and reagents; PB analyzed data and co-wrote the manuscript and JAM designed the study and wrote the manuscript.

## Data availability statement

The authors declare that all data supporting the findings of this study are available in the article along with supplementary Information file.

## Conflict of interest

None of the authors have any conflict of interest.

Please refer to the accompanying ICMJE disclosure forms for further details.

## References

[1] Rehermann B., Thimme R. (2019). Insights from antiviral therapy into immune responses to hepatitis B and C virus infection. Gastroenterology.

[2] Papatheodoridis G.V., Chan H.L., Hansen B.E., Janssen H.L., Lampertico P. (2015). Risk of hepatocellular carcinoma in chronic hepatitis B: assessment and modification with current antiviral therapy. J Hepatol.

[3] Revill P.A., Chisari F.V., Block J.M., Dandri M., Gehring A.J., Guo H. (2019). A global scientific strategy to cure hepatitis B. The Lancet Gastroenterol Hepatol.

[4] Urban S., Schulze A., Dandri M., Petersen J. (2010). The replication cycle of hepatitis B virus. J Hepatol.

[5] Hong X., Kim E.S., Guo H. (2017). Epigenetic regulation of hepatitis B virus covalently closed circular DNA: implications for epigenetic therapy against chronic hepatitis B. Hepatology.

[6] Gilbert S., Galarneau L., Lamontagne A., Roy S., Belanger L. (2000). The hepatitis B virus core promoter is strongly activated by the liver nuclear receptor fetoprotein transcription factor or by ectopically expressed steroidogenic factor 1. J Virol.

[7] Oropeza C.E., Tarnow G., Sridhar A., Taha T.Y., Shalaby R.E., McLachlan A. (2020). The regulation of HBV transcription and replication. Adv Exp Med Biol.

[8] Rappaport A.M. (1958). The structural and functional unit in the human liver (liver acinus). Anat Rec.

[9] Jungermann K., Kietzmann T. (1996). Zonation of parenchymal and nonparenchymal metabolism in liver. Annu Rev Nutr.

[10] Pugh C.W., Ratcliffe P.J. (2017). New horizons in hypoxia signaling pathways. Exp Cell Res.

[11] Palazon A., Goldrath A.W., Nizet V., Johnson R.S. (2014). HIF transcription factors, inflammation, and immunity. Immunity.

[12] Hammond F.R., Lewis A., Elks P.M. (2020). If it's not one thing, HIF's another: immunoregulation by hypoxia inducible factors in disease. FEBS J.

[13] Ko C., Chakraborty A., Chou W.M., Hasreiter J., Wettengel J.M., Stadler D. (2018). Hepatitis B virus genome recycling and de novo secondary infection events maintain stable cccDNA levels. J Hepatol.

[14] Hayer J., Jadeau F., Deleage G., Kay A., Zoulim F., Combet C. (2013). HBVdb: a knowledge database for Hepatitis B Virus. Nucleic Acids Res.

[15] Mühlemann B., Jones T.C., Damgaard PdB., Allentoft M.E., Shevnina I., Logvin A. (2018). Ancient hepatitis B viruses from the Bronze age to the medieval period. Nature.

[16] McNaughton A.L., D'Arienzo V., Ansari M.A., Lumley S.F., Littlejohn M., Revill P. (2019). Insights from deep sequencing of the HBV genome-unique, tiny, and misunderstood. Gastroenterology.

[17] Besarab A., Provenzano R., Hertel J., Zabaneh R., Klaus S.J., Lee T. (2015). Randomized placebo-controlled dose-ranging and pharmacodynamics study of roxadustat (FG-4592) to treat anemia in nondialysis-dependent chronic kidney disease (NDD-CKD) patients. Nephrol Dial Transpl.

[18] Lucifora J., Xia Y., Reisinger F., Zhang K., Stadler D., Cheng X. (2014). Specific and nonhepatotoxic degradation of nuclear hepatitis B virus cccDNA. Science.

[19] Stadelmayer B., Diederichs A., Chapus F., Rivoire M., Neveu G., Alam A. (2020). Full-length 5'RACE identifies all major HBV transcripts in HBV-infected hepatocytes and patient serum. J Hepatol.

[20] EASL (2017). Clinical Practice Guidelines on the management of hepatitis B virus infection. J Hepatol.

[21] Liu P.J., Harris J.M., Marchi E., D'Arienzo V., Michler T., Wing P.A.C. (2020). Hypoxic gene expression in chronic hepatitis B virus infected patients is not observed in state-of-the-art in vitro and mouse infection models. Sci Rep.

[22] Guidotti L.G., Matzke B., Schaller H., Chisari F.V. (1995). High-level hepatitis B virus replication in transgenic mice. J Virol.

[23] Halpern K.B., Shenhav R., Matcovitch-Natan O., Toth B., Lemze D., Golan M. (2017). Single-cell spatial reconstruction reveals global division of labour in the mammalian liver. Nature.

[24] D'Arienzo V., Ferguson J., Giraud G., Chapus F., Harris J.M., Wing P.A.C. (2020). The CCCTC-binding factor CTCF represses hepatitis B virus enhancer I and regulates viral transcription. Cell Microbiol.

[25] Fang B., Mane-Padros D., Bolotin E., Jiang T., Sladek F.M. (2012). Identification of a binding motif specific to HNF4 by comparative analysis of multiple nuclear receptors. Nucleic Acids Res.

[26] Kim K., Boo K., Yu Y.S., Oh S.K., Kim H., Jeon Y. (2017). RORalpha controls hepatic lipid homeostasis via negative regulation of PPARgamma transcriptional network. Nat Commun.

[27] Masson N., Keeley T.P., Giuntoli B., White M.D., Puerta M.L., Perata P. (2019). Conserved N-terminal cysteine dioxygenases transduce responses to hypoxia in animals and plants. Science.

[28] Wilson E.M., Bial J., Tarlow B., Bial G., Jensen B., Greiner D.L. (2014). Extensive double humanization of both liver and hematopoiesis in FRGN mice. Stem Cell Res.

[29] Shalaby R.E., Iram S., Cakal B., Oropeza C.E., McLachlan A. (2017). PGC1alpha transcriptional adaptor function governs hepatitis B virus replication by controlling HBcAg/p21 protein-mediated capsid formation. J Virol.

[30] Wing P.A., Davenne T., Wettengel J., Lai A.G., Zhuang X., Chakraborty A. (2019). A dual role for SAMHD1 in regulating HBV cccDNA and RT-dependent particle genesis. Life Sci Alliance.

[31] Villa P., Arioli P., Guaitani A. (1991). Mechanism of maintenance of liver-specific functions by DMSO in cultured rat hepatocytes. Exp Cell Res.

[32] Elaut G., Henkens T., Papeleu P., Snykers S., Vinken M., Vanhaecke T. (2006). Molecular mechanisms underlying the dedifferentiation process of isolated hepatocytes and their cultures. Curr Drug Metab.

[33] Smythies J.A., Sun M., Masson N., Salama R., Simpson P.D., Murray E. (2019). Inherent DNA-binding specificities of the HIF-1alpha and HIF-2alpha transcription factors in chromatin. EMBO Rep.

[34] D'Arienzo V., Magri A., Harris J.M., Wing P.A.C., Ko C., Rubio C.O. (2019). A PCR assay to quantify patterns of HBV transcription. J Gen Virol.

[35] Zhang X., Lu W., Zheng Y., Wang W., Bai L., Chen L. (2016). In situ analysis of intrahepatic virological events in chronic hepatitis B virus infection. J Clin Invest.

[36] Wooddell C.I., Yuen M.F., Chan H.L., Gish R.G., Locarnini S.A., Chavez D. (2017). RNAi-based treatment of chronically infected patients and chimpanzees reveals that integrated hepatitis B virus DNA is a source of HBsAg. Sci Transl Med.

[37] Rivkina M.B., Lunin V.G., Mahov A.M., Tikchonenko T.I., Kukain R.A. (1988). Nucleotide sequence of integrated hepatitis B virus DNA and human flanking regions in the genome of the PLC/PRF/5 cell line. Gene.

[38] Su T.S., Hwang W.L., Yauk Y.K. (1998). Characterization of hepatitis B virus integrant that results in chromosomal rearrangement. DNA Cell Biol.

[39] Turton K.L., Stephenson V., Badmalia M.D., Coffin C.S., Patel T.R. (2020). Host transcription factors in hepatitis B virus RNA synthesis. Viruses.

[40] Hinojosa-Laborde C., Mifflin S.W. (2005). Sex differences in blood pressure response to intermittent hypoxia in rats. Hypertension.

[41] Rubin B.R., Milner T.A., Pickel V.M., Coleman C.G., Marques-Lopes J., Van Kempen T.A. (2020). Sex and age differentially affect GABAergic neurons in the mouse prefrontal cortex and hippocampus following chronic intermittent hypoxia. Exp Neurol.

[42] Fuady J.H., Gutsche K., Santambrogio S., Varga Z., Hoogewijs D., Wenger R.H. (2016). Estrogen-dependent downregulation of hypoxia-inducible factor (HIF)-2alpha in invasive breast cancer cells. Oncotarget.

[43] Wang S.H., Yeh S.H., Lin W.H., Yeh K.H., Yuan Q., Xia N.S. (2012). Estrogen receptor alpha represses transcription of HBV genes via interaction with hepatocyte nuclear factor 4alpha. Gastroenterology.

[44] Shimizu I., Kohno N., Tamaki K., Shono M., Huang H.W., He J.H. (2007). Female hepatology: favorable role of estrogen in chronic liver disease with hepatitis B virus infection. World J Gastroenterol.

[45] Das T., Soren K., Yerasi M., Kamle A., Kumar A., Chakravarty S. (2020). Molecular basis of sex difference in neuroprotection induced by hypoxia preconditioning in zebrafish. Mol Neurobiol.

[46] Ploumakis A., Coleman M.L. (2015). OH, the places you'll go! Hydroxylation, gene expression, and cancer. Mol Cell.

[47] Hallez C., Li X., Suspène R., Thiers V., Bouzidi M.S., Dorobantu C.M. (2019). Hypoxia-induced human deoxyribonuclease I is a cellular restriction factor of hepatitis B virus. Nat Microbiol.

[48] Budzinska M.A., Shackel N.A., Urban S., Tu T. (2018). Sequence analysis of integrated hepatitis B virus DNA during HBeAg-seroconversion. Emerg Microbes Infect.

[49] Jiang Z., Jhunjhunwala S., Liu J., Haverty P.M., Kennemer M.I., Guan Y. (2012). The effects of hepatitis B virus integration into the genomes of hepatocellular carcinoma patients. Genome Res.

[50] Maini M.K., Pallett L.J. (2018). Defective T-cell immunity in hepatitis B virus infection: why therapeutic vaccination needs a helping hand. The Lancet Gastroenterol Hepatol.

[51] Xiong X.X., Qiu X.Y., Hu D.X., Chen X.Q. (2017). Advances in hypoxia-mediated mechanisms in hepatocellular carcinoma. Mol Pharmacol.

[52] Xia Y., Liang T.J. (2019). Development of direct-acting antiviral and host-targeting agents for treatment of hepatitis B virus infection. Gastroenterology.

[53] Farquhar M.J., Humphreys I.S., Rudge S.A., Wilson G.K., Bhattacharya B., Ciaccia M. (2017). Autotaxin-lysophosphatidic acid receptor signalling regulates hepatitis C virus replication. J Hepatol.

[54] Wilson G.K., Brimacombe C.L., Rowe I.A., Reynolds G.M., Fletcher N.F., Stamataki Z. (2012). A dual role for hypoxia inducible factor-1alpha in the hepatitis C virus lifecycle and hepatoma migration. J Hepatol.

[55] Vassilaki N., Kalliampakou K.I., Kotta-Loizou I., Befani C., Liakos P., Simos G. (2013). Low oxygen tension enhances hepatitis C virus replication. J Virol.

[56] Morinet F., Casetti L., Francois J.H., Capron C., Pillet S. (2013). Oxygen tension level and human viral infections. Virology.

[57] Testoni B., Lebosse F., Scholtes C., Berby F., Miaglia C., Subic M. (2019). Serum hepatitis B core-related antigen (HBcrAg) correlates with covalently closed circular DNA transcriptional activity in chronic hepatitis B patients. J Hepatol.

[58] Sanghani N.S., Haase V.H. (2019). Hypoxia-inducible factor Activators in renal anemia: current clinical experience. Adv Chronic Kidney Dis.

