# Supplementary material for: Hypoxia inducible factors regulate hepatitis B virus replication by activating the basal core promoter
Source: J Hepatol. 2021 Jul;75(1):64–73. doi: 10.1016/j.jhep.2020.12.034 (PMC8214165; doi:10.1016/j.jhep.2020.12.034)
Supplement: Multimedia component 1 [file mmc1.pdf]

Hypoxia inducible factors regulate hepatitis B virus replication by activating the basal core promoter

Peter AC Wing, Peter Jianrui Liu, James M Harris, Andrea Magri, Thomas Michler, Xiaodong Zhuang, Helene Borrmann, Rosalba Minisini, Nicholas R Frampton, Jochen Wettengel, Laurent Mailly, Valentina D'Arienzo, Tobias Riedl, Luis Nobre, Michael Weekes, Mario Pirisi, Mathias Heikenwelder, Thomas F Baumert, Ester Hammond, David R Mole, Ulrike Protzer, Peter Balfe, Jane A McKeating

Table of contents

Supplementary materials and methods..2

Supplementary figures..11

Supplementary references..17

Supplementary materials and methods

Primary Human Hepatocyte culture. Cryopreserved PHH from Thermofisher where defrosted and plated on collagen coated 24 well plates in Williams E Media (no Phenol Red) supplemented with hepatocyte plating supplement pack (CM3000 Thermofisher) and 2% DMSO. Cells were left to adhere for 16h and the media changed to hepatocyte maintenance media (CM4000 Thermofisher) supplemented with 2% DMSO, incubated for a further 72h and incubated in normoxic or hypoxic conditions as described. Cells were re-perfused with maintenance media every 48-72h until harvesting for RNA extraction.

Human and non-human HBV phylogeny. The HBV Database (http://hbvdb.ibcp.fr) [1] was searched for the HRE motif RCGTGC and the number and location of matches enumerated. The Neighbor-joining phylogenetic tree (**Fig.1a**) is based on that of McNaughton and colleagues [2] and was built from an alignment of complete (~3.2Kb) NCBI referent genomes of human and non-human HBV with the following accession numbers: Anatidae (Geese): Snow goose; NC_005950.1, Duck; NC_001344.1, Sheldgoose; NC_005890.1 and Ross's goose; NC_005888.1. Other birds: Heron; NC_001486.1, Tinamou; NC_035210.1 and Parrot; NC_016561.1. Other species: Tibetan frog; NC_030446.1, White sucker fish; NC_027922.1 and Bluegill fish; NC_030445.1. Bats: Tent-making bat; NC_024445.1, Horseshoe bat; NC_024444.1, Roundleaf bat; NC_024443.1 and Long-fingered bat; NC_020881.1. New world monkeys: Woolly monkey; NC_028129.1 and Capuchin Monkey; KY703886. Other Mammals: Woodchuck; M19183.1 and Ground squirrel; NC_001484.1. Old World Primates: Orangutan; F193863, Gorilla; FJ798097 and Chimpanzee; FJ798098. Human: Genotype (Gt) A; EU054331, X02763, Gt B; AB219428, D00331 Gt C; GQ924620, GQ358158, Gt D; AF121240, FJ904433, Gt E; AB106564, AY738145, Gt F; AY090458, X75658, Gt G; AB064313, AF160501 and Gt H; AY090454, FJ356716 (HBV sequences are referents from the HBV database[1]).

Liver biopsy samples. Liver biopsies from chronic HBV infected (n=19) and non-infected subjects (n=8) were collected with informed consent to have a small part of the biopsy specimen, exceeding that needed for complete pathology examination, saved for research purposes. A small fragment of the biopsy specimen was immersed in RNAlater and saved at -80°C for further analyses. The local ethical committee approved the use of this archival material (CE90/19) together with anonymized clinical and demographic data for the purposes

of this study. RNA was isolated and concentration and integrity assessed by Tapestation 2200 (Agilent Technologies).

nCounter analysis of HBV and host hypoxia transcripts. HBV probe sequence was designed to target the 3' of overlapping HBV RNA species; target sequence:
CTAGGCTGTGCTGCCAACTGGATCCTGCGCGGGACGTCCTTTGTTTACGTCCCGTCGGCGCTGAATCC CGCGGACGACCCTTCTCGGGGCCGCTTGGGAC.
Specificity of the probe was determined by setting the lower detection limit at the median + 1.5 x Interquartile range (14.0685) of the non-infected controls (n=8). 18/19 patients had detectable HBV RNA by nCounter quantification. All patients were grouped based on their expression of HBV RNAs into low or high based on deviation from the median expression. Transcript levels of fifteen selected HIF-regulated genes: *BNIP3*, Bcl2 interacting protein 3; *BNIP3L*, Bcl2/adenovirus e1b 19kDa protein-interacting protein 3-like; *CAIX*, Carbonic anhydrase IX; *EPO*, Erythropoietin; *FGF11*, Fibroblast growth factor 11; *FAM115C,* TRPM8 channel associated factor 2 ; *GLUT1,* Glucose transporter 1; I*GLON*5, Iglon family member 5; *LCN15*, Lipocalin 15; *LOX*, Lysyl oxidase; *NDRG1*, n-Myc downstream regulated 1; *PFKFB4*, 6-phosphofructo-2-kinase/fructose-2,6-biphosphatase 4; *SPAG4*, Sperm associated antigen 4 and *TNS1*, Tensin 1; VEGFA, Vascular endothelial growth factor alpha, as previously described in[3]) were quantified by nCounter (NanoString).

HBV reporter virus and quantifying Gaussia luciferase. HBV reporter virus expressing Gaussia luciferase (rHBV-GLuc) was generated as previously reported [4]. HepG2-NTCP cells infected with the HBV-Gluc reporter virus were maintained for 72h in either 18% or 1% oxygen. Cells were removed from the hypoxia chamber and supernatants clarified by centrifugation, these were then mixed 1:1 with 500µg/ml Coelenterazine (PJK, Germany) and luciferase activity measured on a FluoSTAR Omega (BMG).

Ad-HBV1.3 transduction of HepG2-NTCP cells. HepG2-NTCP cells were seeded to 80% confluency before transduction with Ad-HBV-GFP at an MOI of 20 ffu/cell. Adenoviral vector was removed after 2h and cells transferred to 18% or 1% oxygen for 72h. Extracellular HBV DNA was quantified by qPCR as described below. To account for transduction efficiency, HBV particle production measurements were normalized to quantification of GFP fluorescence.

HBV promoter assay. HepG2-NTCP cells were transfected using Fugene-6 (Promega) with pGL3-HBV-Luciferase (provided by Wang-Shick Ryu, Yonsei University, South Korea) [5] or pGL3-HRE-Luciferase promoter constructs (provided by Margret Ashcroft, University of Cambridge, UK) and 24h later cultured under 18% or 1% oxygen with samples harvested at the stated times. Luciferase activity in cell lysates were quantified with a Luciferase assay (Promega) using a Centro LB960 Microluminometer.

PCR quantification of HBV DNA, RNA and cellular transcripts. Total DNA and RNA were extracted from cells using the All-Prep-DNA-RNA kit (Qiagen) and PCR of cccDNA performed as described[6]. Briefly, DNA was treated with 5U of T5 exonuclease (NEB) for 37°C for 30 minutes then heat inactivated at 95°C. Samples were then amplified in a SYBR green qPCR (PCR Biosystems) (See CTAT Table). RNA was treated with on-column DNase I (Qiagen), with 0.5-1µg used for cDNA synthesis (PCR Biosystems). Host target genes were amplified (See CTAT Table) and HIF target genes quantified using TaqMan probes. Quantification of HBV pC/pgRNA, preS1, preS2/S and HBx transcripts was as previously reported [7]. All PCRs were performed on a Lightcycler 96 (Roche).

siRNA knockdown of HIF-1α and HIF-2α. HepG2-NTCP cells were trypsinized and mixed with 50nM of either scramble or HIF-1α/HIF-2α siRNA (See CTAT Table) complexed with DharmaFECT4 transfection reagent (Dharmacon) according to manufacturer's instructions. Transfected cells were seeded and left to recover for 4h after which media was replenished. Cells were infected with HBV at an MOI of 200 24h post siRNA delivery for 6h then transferred to either 18% or 1% oxygen for 72h after which cells were harvested in RLT buffer (Qiagen) for subsequent PCR analysis.

siRNA delivery into HBV transgenic mice and immunostaining liver sections. Animal experiments were conducted in accordance with the regulations of the Society for Laboratory Animal Science (GV-SOLAS) and the Federation of Laboratory Animal Science Associations (FELASA) and approved by the local Animal Care and Use Committee of Upper Bavaria and followed 3R rules. HBVtg mice (strain HBV1.3.32) [8, 9] carrying a 1.3-fold overlength HBV genome (genotype D) on a C57BL/6J background were used for all experiments. Mice were between 12-15 weeks old and groups contained equal numbers of males and females. HIF-1β siRNAs (See CTAT Table) were complexed with Invivofectamine 3.0 reagent (ThermoFisher)

before tail vein injection (1 µg/g body weight). HBeAg was quantified from mouse sera after using Architect HBsAg Manual Diluent (Ref: 6C32-27) on an Architect™ platform (Abbott, Germany). Immediately after sacrifice, 0.4x1-1.5 cm pieces of liver were placed in 500µL RNA later and stored at -20ºC. Tissue was homogenized (TissueLyser LT (Qiagen), 5 min, 50 Hz) and RNA prepared using an RNeasy Mini kit (Qiagen). Livers were fixed in 4% buffered formalin for 48 h, dehydrated and embedded in paraffin. Two µm-thin sections were stained with a rabbit anti-HBV core antibody (OriGene technologies Inc.; Rockville, MD, USA; CAT#: AP10430PU-N) on a Leica Bond MAX system (Leica Biosystems, Nussloch, Germany). Tissue slides were scanned using a SCN 400 slide scanner (Leica Biosysstems) and analyzed by two experienced scientists in a blinded manner. Criteria included strength of HBc expression (scale: 0 (none) to 3 (very strong)) as well as zonal distribution (scale: 0 (equal distribution throughout liver zones) to 2 (strong pericentral distribution).

HBV infected human liver chimeric mice. FRG-NOD mice were housed and bred at the INSERM U1110 animal facility (regional agreement n° E-67- 482-7) and fed 2-(2-nitro-4-trifluoromethylbenzoyl)-1,3-cyclohexanedione (NTBC) in their drinking water at 16 mg/L. Six-week-old FRG-NOD mice received 1.5x109 pfu of an adenoviral vector encoding for the urokinase-like plasminogen activator and fed NTBC at 8 mg/L. Forty eight hours later mice were intrasplenically transplanted with 106 cryopreserved PHHs (Life technologies) and fed NTBC at 0.8 mg/L. During the following days NTBC concentration was decreased every 2 days to 0.4 mg/L and 0.2 mg/L, and finally withdrawn. Efficient transplantation was assessed 8 weeks later by measuring human albumin levels in mouse serum by a specific ELISA (Bethyl). The transplantation procedure was approved by the local ethic committee and authorized by the French ministry of research and higher education. Successfully transplanted mice were infected with 10^9 HBV genome equivalents purified from the concentrated supernatant of the HepG2.2.15 cell line (HBV genotype D subtype ayw) and four weeks later the animals were sacrificed and 0.4x1-1.5 cm^2 pieces of liver were snap frozen for preparing chromatin as detailed below.

Preparation of chromatin and ChIP. $1x10^7$ HepG2-pEPI cells were harvested from 15 cm plates as previously reported [10], or HBV infected human liver chimeric mouse tissue samples were thawed and homogenized. Homogenized cells or tissue samples were fixed with 1% formaldehyde (Sigma, 47608) for 10 min (r/t) before quenching with 125mM glycine. Cells

were washed twice with ice cold PBS, pelleted (800rpm, 10 minutes 4°C) and lysed in 500uL of Nuclear Extraction buffer (10 mM Tris-HCl (pH 8.0), 10 mM NaCl, 1% NP-40) supplemented with protease inhibitor cocktail (Roche). Samples diluted 1:1 in ChIP Dilution Buffer (0.01% SDS, 1.1% Triton, 0.2mM EDTA; 16.7 mM Tris pH8.1, 167mM NaCl) and pulse sonicated (Bioruptor®, Diagenode) at high power for 30 min at 4°C (15s on/15s off). After centrifugation (1300rpm, 10 min) lysates were immunoprecipitated overnight with 2–5 µg of antibody of interest or irrelevant IgG control (See CTAT Table), then pulled down with Protein agarose beads (Millipore, 16-156). Precipitates were washed in low salt buffer (0.1% SDS, 1% Triton, 2mMEDTA, 20mM Tris pH8.1, 150mM NaCl), high salt buffer (0.1% SDS, 1% Triton, 2mM EDTA, 20mM Tris pH 8.1, 500mM NaCl), LiCl Buffer (1% Igepal, 1mM EDTA, 10mM Tris pH 8.1, 250mM LiCl, 1% sodium deoxycholate) and finally twice in TE buffer (10mM Tris pH8.0, 1mM EDTA) before being eluted from the beads in 240uL of elution buffer (0.1M $NaHCO_3$, 1% SDS). Complexes were reverse crosslinked in a heated shaker at 65°C overnight, 1400rpm, in the presence of 200 mM NaCl. Eluates were treated with Proteinase K (SIGMA) and RNaseA (SIGMA) before cleanup using MiniElute PCR Purification columns. Samples were amplified using SYBR green qPCR (PCR Biosystems, UK) on a LightCycler96 (Roche) and % Input calculated for each sample or IgG relative to input controls, if the % Input of the sample was not greater than that of the matched IgG they were excluded from the analysis.

SDS–PAGE and western blots. The cells were lysed in RIPA buffer (20 mM Tris, pH 7.5, 2 mM EDTA, 150 mM NaCl, 1% NP40, and 1% sodium deoxycholate) supplemented with protease inhibitor cocktail tablets (Roche). 4× reducing buffer was added to samples before incubating at 95°C for 5 min. Proteins were separated on a 10% polyacrylamide gel and transferred to PVDF membranes (Amersham). Membranes were blocked in PBST, 5% skimmed milk (Sigma), and proteins detected using specific primary and HRP-secondary antibodies. Protein bands were detected using Pierce SuperSignal West Pico chemiluminescent substrate kit (Pierce) and images collected with a PXi Touch Imaging system (Syngene).

Accession codes. RNA-Seq and ChIP-Seq datasets are available for download from Gene Expression Omnibus (GSE120885, GSE120886 and GSE120887). The mass spectrometry proteomics data have been deposited to the ProteomeXchange Consortium via PRIDE [11] partner repository with the dataset identifier PXD020086.

Cell Cycle FACS. Following hypoxic treatment with respective agents, cells were incubated in 10uM bromodeoxuridine (BrdU) (Sigma) for 30 minutes. Cells were trypsinized and washed thrice in PBS, before fixation in ice cold 70% Ethanol for 20 minutes. Cells were incubated in warmed pepsin solution (Sigma) for 30 minutes, then pelleted and treated with 2M HCl. Cells were washed in PBS and blocked (0.5% BSA/0.5%Tween) for 30 minutes. Cells were stained with aBrdU488 antibody (Biolegend), and then with propidium iodide (Invitrogen) each for 30 minutes. Samples were then acquired on a CyAn flow cytometer.

Quantification of extracellular HBV DNA. Supernatants from either HepG2.2.15 or Ad-HBV transduced HepG2-NTCP cells were first DNase digested with 1U of DNase at 37ºC for 30 minutes (Thermofisher, UK) to remove any non-virion associated HBV DNA. A 2X lysis buffer containing 0.1M TrisHCl pH7.4, 50mM KCl, 0.25% Triton X-100 and 40% glycerol was added to the digested supernatants and heated at 65ºC for 10 minutes. In activated lysates were diluted 1:2 with molecular grade water for qPCR analysis to detect HBV rcDNA using specific primers HBV4F and HBV4R. Copy numbers were calculated using a dilution series of an external HBV plasmid.

Tandem Mass Tag (TMT) based whole cell lysate proteomics. Huh-7 cells were washed twice with PBS and lysed in 150 µl of 6M Guanidine/50 mM HEPES pH 8.5. Samples were vortexed extensively then sonicated. Cell debris was removed by centrifuging at 13,000 g for 10 min twice. Dithiothreitol (DTT) was added to a final concentration of 5 mM and samples were incubated for 20 min. Cysteines were alkylated with 15 mM iodoacetamide and incubated 20 min at room temperature in the dark. Excess iodoacetamide was quenched with DTT for 15 min. Samples were diluted with 200 mM HEPES pH 8.5, 1.5 M Guanidine followed by digestion at room temperature for 3 hr with LysC protease at a 1:100 protease-to protein ratio. Trypsin was then added at a 1:100 protease-to-protein ratio followed by overnight incubation at 37°C. The reaction was quenched with 1 % formic acid, samples were spun at 21,000 g for 10 min to remove debris and undigested protein, then subjected to C18 solid-phase extraction (Sep-Pak, Waters) and vacuum centrifuged to near-dryness. In preparation for TMT labelling, desalted peptides were dissolved in 200 mM HEPES pH 8.5. Peptide concentration was measured by micro BCA (Pierce), and 50 µg of peptide per sample was labelled with TMT reagent (control - TMT 126; hypoxia - TMT 127N). TMT reagents (0.8 mg) were dissolved in 43 µL anhydrous acetonitrile and 5 µl added to peptide sample at a final acetonitrile concentration of 30 % (v/v).

Following incubation at room temperature for 1 hr, the reaction was quenched with hydroxylamine to a final concentration of 0.5 % (v/v). TMT-labelled samples were combined at a 1:1 ratio. The sample was vacuum-centrifuged to near dryness and subjected to C18 solid-phase extraction (SPE) (Sep-Pak, Waters). Offline high pH reversed-phase fractionation of peptides was performed, with peptide fractions combined as described previously [12].

Liquid Chromatography and Tandem Mass Spectrometry. An unfractionated singleshot was analysed initially to ensure similar peptide loading for both TMT channels, thus avoiding the need for excessive electronic normalization. As the sum signal:noise for the two channels was very similar, data from the unfractionated singleshot experiment was analysed with data for the corresponding fractions to increase the overall number of peptides quantified. Mass spectrometry data were acquired using an Orbitrap Elite mass spectrometer (unfractionated singleshot) coupled with a Proxeon EASY-nLC II liquid chromatography (LC) pump or an Orbitrap Fusion (fractions) coupled with a Proxeon EASY-nLC 1000 LC pump (Thermo Fisher Scientific, San Jose, CA). Each analysis used an MS3-based TMT method [13, 14].

Orbitrap Elite Experiments. Peptides were separated on a 100 mm inner diameter microcapillary column packed with 0.5 cm of Magic C4 resin (5 mm, 100 Å, Michrom Bioresources) followed by approximately 20 cm of Maccel C18 resin (3 mm, 200 Å, Nest Group). Peptides were separated using a 3 hr gradient of 6 to 30 % acetonitrile in 0.125 % formic acid at a flow rate of 300 nl/min. The scan sequence began with an MS1 spectrum (Orbitrap analysis, resolution 60,000, 300-1500 Th, AGC target $1x10^6$, maximum injection time 150 ms). The top ten precursors were then selected for MS2/MS3 analysis. MS2 analysis consisted of CID (quadrupole ion trap analysis, AGC $2x10^3$, NCE 35, q-value 0.25, maximum injection time 100 ms). MS3 precursors were fragmented by HCD prior to Orbitrap analysis (NCE 50, max AGC $1.5x10^5$, maximum injection time 250 ms, isolation specificity 2.5 Th, resolution 30,000) [13].

Orbitrap Fusion Experiments. Peptides were separated on a 75 mm inner diameter microcapillary column packed with 0.5 cm of Magic C4 resin (5 mm, 100 Å, Michrom Bioresources) followed by approximately 20 cm of GP118 resin (1.8 mm, 120 Å, Sepax Technologies). Peptides were separated using a 3 hr gradient of 6 to 30 % acetonitrile in 0.125 % formic acid at a flow rate of 300 nl/min. The scan sequence began with an MS1 spectrum

(Orbitrap analysis, resolution 120,000, 400-1400 Th, AGC target $2x10^5$, maximum injection time 200 ms). 'Top speed' (2 s) was selected for MS2 analysis, which consisted of CID (quadrupole ion trap analysis, AGC $4x10^3$, NCE 35, maximum injection time 150 ms). The top ten precursors were selected for MS3 analysis, in which precursors were fragmented by HCD prior to Orbitrap analysis (NCE 55, max AGC $5x10^4$, maximum injection time 250 ms, isolation specificity 0.5 Th, resolution 60,000) [13].

Mass spectrometry data analysis. Mass spectra were processed using a Sequest-based in-house software pipeline as described previously[12]. Briefly, MS spectra were converted to mzXML using a modified version of ReAdW.exe. A combined database was constructed from a combined human Uniprot and Trembl database (February 4th, 2014) and common contaminants such as porcine trypsin and endoproteinase LysC removed. The combined database was concatenated with a reverse database composed of all protein sequences in reversed order. Searches were performed using a 20 ppm precursor ion tolerance. Product ion tolerance was set to 0.03 Th. TMT tags on lysine residues and peptide N termini (229.162932 Da) and carbamidomethylation of cysteine residues (57.02146 Da) were set as static modifications, while oxidation of methionine residues (15.99492 Da) was set as a variable modification. To control the fraction of erroneous protein identifications, we used a target-decoy strategy [15, 16].

Peptide spectral matches (PSMs) were filtered to an initial peptide-level false discovery rate (FDR) of 1% with subsequent filtering to attain a final protein-level FDR of 1%. PSM filtering was performed using a linear discriminant analysis, as described previously [17], considering the following parameters: XCorr, DCn, missed cleavages, peptide length, charge state, and precursor mass accuracy. Protein assembly was guided by principles of parsimony to produce the smallest set of proteins necessary to account for all observed peptides. Proteins were quantified by summing TMT reporter ion counts across all matching peptide-spectral matches using in-house software. Briefly, a 0.003 Th window around the theoretical m/z of each reporter ion (126, 127N) was scanned for ions, and the maximum intensity nearest to the theoretical m/z was used. We required every individual peptide used for quantitation to contribute sufficient TMT reporter ions (minimum of 1,250 per spectrum) so that each on its own provided a representative picture of relative protein abundance [13]. We additionally employed an isolation specificity filter to minimize peptide co-isolation [14]. Peptide-spectral

matches with poor quality MS3 spectra (more than 9 TMT channels missing and/or a combined signal:noise ratio of less than 100 across all TMT reporter ions) or no MS3 spectra at all were excluded from quantitation. Protein quantitation values were exported for further analysis in Excel. Reverse and contaminant proteins were removed, then both reporter ion channel was summed across all quantified proteins and normalized assuming equal protein loading across samples. Data are available via ProteomeXchange with identifier PXD020086.

Statistics. All experiments were performed at least three times and detailed replicate numbers are provided in figure legends. *p*-values were determined using: ANOVA analysis for more than two group comparisons, Mann-Whitney U-Test (two group comparisons; unpaired data) or Wilcoxon matched-pairs signed rank test (two group comparison; paired data) using PRISM version 8. In the figures ∗ denotes $p < 0.05$, ∗∗ denotes $p < 0.01$, ***denotes $p < 0.001$, ****denotes $p < 0.0001$.

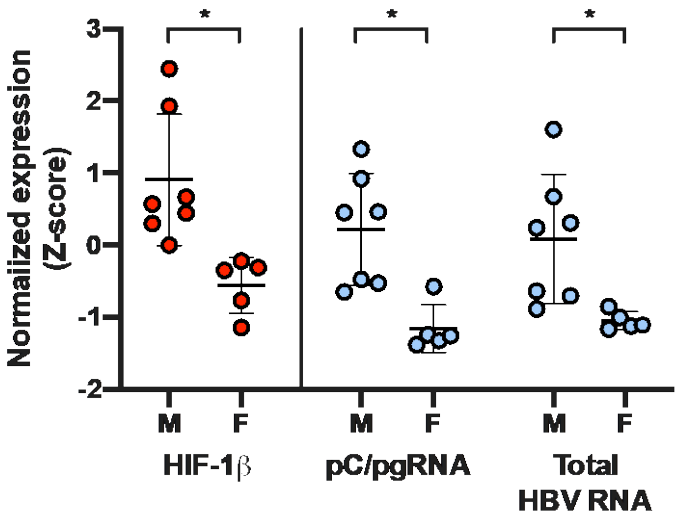

Fig. S1. HIF-1β silencing efficacy in male and female HBV1.3 transgenic mice. Transcript levels of HIF-1b, pC/pgRNA and total HBV RNAs were measured by qPCR and aΔCt was calculated for the siHIF1b treated animals relative to the siCtrl. The mean ΔCt for all three parameters was used to compute Z-scores, enabling a direct comparison of the three transcripts. Stratification of the Z-scores by sex shows a significant effect of HIF-1β silencing in male mice compared to females (* $p < 0.05$, student's t-test with Bonferroni multiple correction).

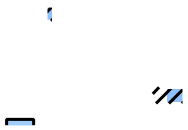

Fig. S2. Hypoxia increases extracellular HBV DNA.

HepG2-NTCP cells transduced with Ad-HBV or HepG2.2.15 cells were incubated at either 18% or 1% oxygen for 72h. Extracellular HBV DNA was quantified by qPCR and expressed relative to the 18% control. Data is plotted as mean ± SD and derived from 3 independent experiments. Statistical analysis was performed using a Mann Whitney U Test (** = $p \leq 0.01$)

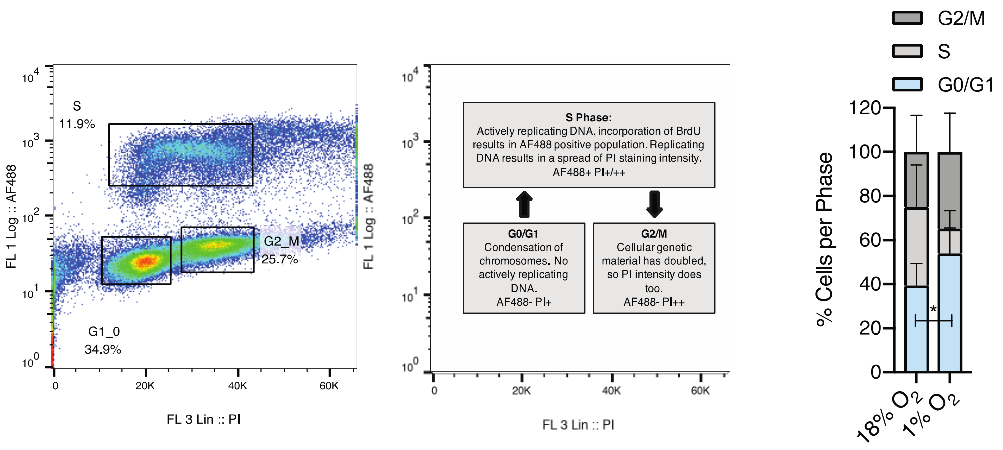

Fig. S3. Cell cycle analysis of HepG2-NTCP cells cultured at 18% or 1% oxygen.

(a) A representative FACs plot showing HepG2-NTCP cells cultured at 18% oxygen, following staining with anti-BrdU-AF488 and propidium iodide. Adjacent schematic describes the gating strategy applied to ascribe cell cycle phase to the population. **(b)** HepG2-NTCP cells treated for 72h with 18% or 1% oxygen were fixed for FACS staining. The relative proportion of cells in each phase of the cell cycle were determined by quantification of BrdU incorporation, and propidium iodide staining by flow cytometry. Data is represented as mean ± SD of 3 independent experiments consisting of at least 3 replicates per condition.

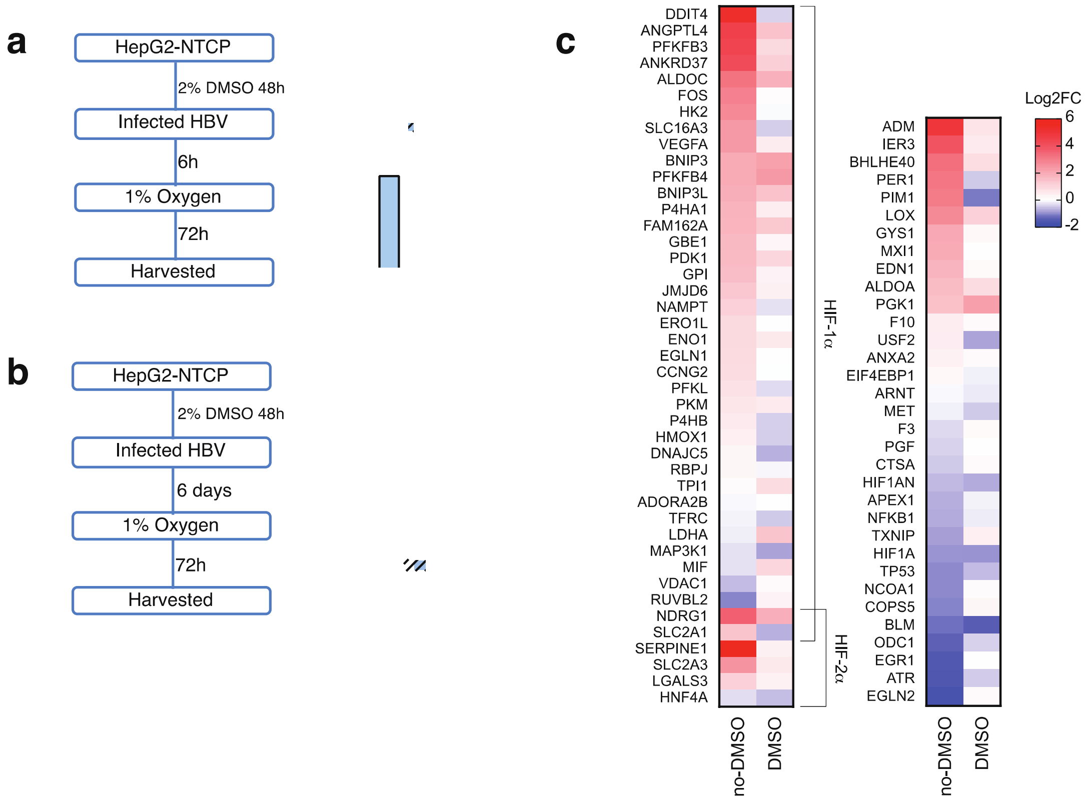

Fig. S4. DMSO blunts the cellular response to low oxygen.

(**a**) HepG2-NTCP cells were treated with 2% DMSO for three days, inoculated with HBV (MOI 200) for 6h and cultured under 18% or 1% oxygen for 72h. RNA was isolated and pC/pgRNA levels quantified by PCR, data is presented relative to 18% oxygen. (**b**) HepG2-NTCP cells were treated with 2% DMSO as before, infected with HBV (MOI 200) for 6 days and then cultured under 18% or 1% oxygen for 72h. RNA was isolated and HBV pC/pgRNA levels quantified by PCR, data is presented relative to 18% oxygen. Data represent the mean ± SD derived from 3 independent experiments and statistical analysis performed using a Mann-Whitney U-Test. (* $p \leq 0.05$). (**c**) Untreated or DMSO treated HepG2-NTCP cells were cultured under 18% or 1% oxygen conditions for 24h, RNA extracted and transcript levels of 84 hypoxic genes quantified by Qiagen RT2 PCR array. Gene expression was adjusted to 5 referent housekeeping genes and log_2 fold change (Log2FC) calculated relative to their respective 18% oxygen controls. 9 genes were excluded as expression was beneath the limit of detection. Genes were segregated based on HIF-1α and HIF-2α ChIP-Seq, where significant binding (1.5-fold greater RPKM than HIF-1β control) of HIF isoforms was detected in 43 genes. Genes were ranked by FC in no-DMSO samples.

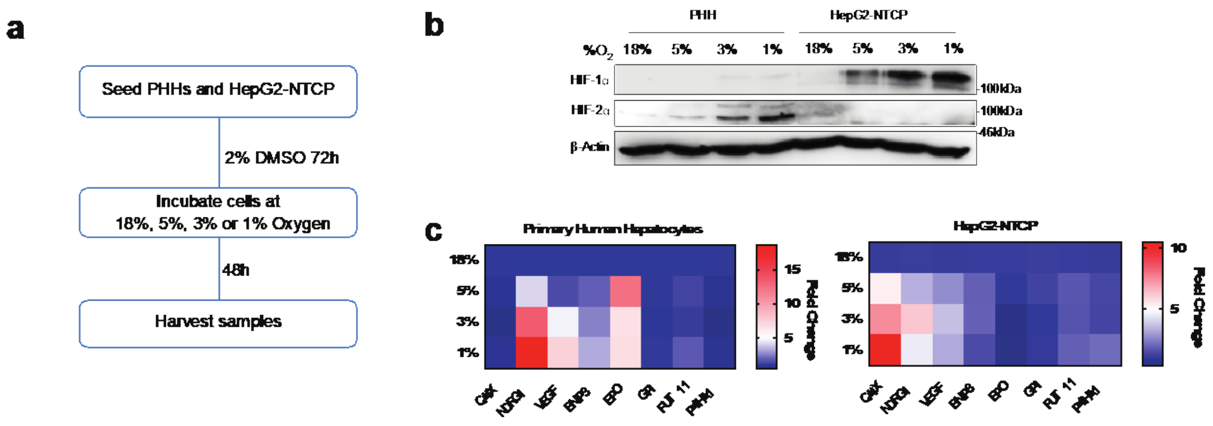

Fig. S5. Hypoxic response of primary human hepatocytes.

(a) Cryopreserved primary human hepatocytes (PHHs) were thawed and cultured in maintenance medium containing 2% DMSO for 72h prior to culturing at variable oxygen tension for 48h. HepG2-NTCP were set up in parallel and treated in a similar manner to the PHH cultures and samples harvested 48h later. (**b**) HIF-1α and HIF-2α expression was assessed by western blot of cell lysates harvested from the indicated oxygen tensions. **(c)** Expression of a panel of HIF responsive genes from both cell types was quantified by PCR. Data is presented in a heatmap as FC of each gene relative to the 18% oxygen control.

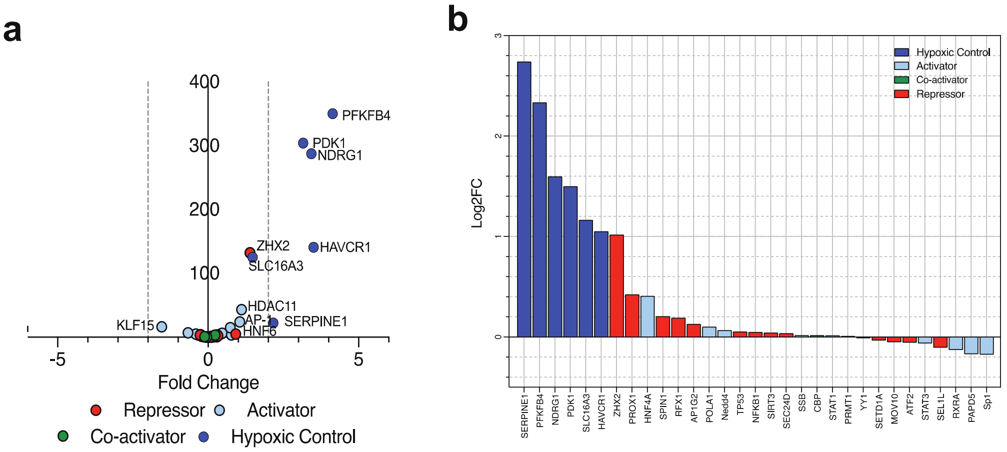

c

Activators		Repressors	
AP-1	Jun proto-oncogene	AP1G2	Adaptor related protein complex 1 subunit gamma 2
C/EBP	CCAAT enhancer binding protein beta	APOBEC3G	Apolipoprotein B mrna editing enzyme catalytic subunit 3G
CREB	CAMP responsive element binding protein 1	ATF2	Activating transcription factor 2
FOXM1	Forkhead box m1	BCL6	BCL6 transcription repressor
GTF2B	General transcription factor IIB	DNASE1	Deoxyribonuclease 1
HNF4A	Hepatocyte nuclear factor 4 alpha	ESR1	Estrogen receptor 1
KLF15	Kruppel like factor 15	HDAC11	Histone deacetylase 11
NEDD4	NEDD4 E3 ubiquitin protein. Ligase	HOXA10	Homeobox A10
NFYB	Nuclear transcription factor Y subunit beta	ID1	Inhibitor of DNA binding 1, HLH protein
NR1H4	Nuclear receptor subfamily 1 group H member 4	MAP3K1	Mitogen-activated protein kinase kinase kinase 1
NR5A2	Nuclear receptor subfamily 5 group A member 2	MOV10	MOV10 risc complex RNA helicase
NRF1	Nuclear respiratory factor 1	NFKB1	Nuclear factor kappa B subunit 1
PAPD5	Terminal nucleotidyltransferase 4B	NR0B2	Nuclear receptor subfamily 0 group B member 2
PAPD7	Terminal nucleotidyltransferase 4A	NR2F2	Nuclear receptor subfamily 2 group F member 2
POLA1	DNA polymerase alpha 1, catalytic subunit	OAS2	2'-5'-Oligoadenylate synthetase 2
POU2F1	POU class 2 homeobox 1	ONECUT1	One cute homeobox 1
PPARA	Peroxisome proliferator activated receptor alpha	PDE4A	Phosphodiesterase 4A
RXRA	Retinoid X receptor alpha	PROX1	Prospero homeobox 1
SP1	Sp1 transcription factor	RFX1	Regulatory factor X1
STAT3	Signal transducer and activator of transcription 3	SEC24D	SEC24 Homolog D, COPII coat complex component
TBP	TATA-box binding protein	SEL1L	SEL1 adaptor subunit of ERAD E3 ubiquitin ligase
**Co-activators**		SETD1A	SET domain containing 1A, histone lysine methyltransferase
CBP	CREB binding protein	SIRT3	Sirtuin 1
PGC1A	PPARG coactivator 1 alpha	SNAI2	Snail family transcriptional repressor 2
PRMT1	Protein arginine methyltransferase 1	SOX7	SRY-Box transcription factor 7
SRC1	Nuclear receptor coactivator 1	SPIN1	Spindlin 1
**Hypoxic Controls**		SSB	Small RNA binding exonuclease protection factor la
HAVCR1	Hepatitis A virus cellular receptor 1	STAT1	Signal transducer and activator of transcription 1
SLC16A3	Monocarboxylic acid transporter 4	SUV39H1	Suppressor of variegation 3-9 homolog 1
PDK1	Pyruvate deydrogenase kinase 1	TNFSF2	Tumour necrosis factor
NDRG1	N-myc downstream regulated 1	TP53	Tumour protein P53
PFKFB4	6-phosphofructo-2-kinase/fructose-2,6-bisphosphatase 4	TP73	Tumour protein P73
SERPINE1	Serpin family E member 1	TSPYL2	TSPY like 2
		YY1	YY1 transcription factor
		ZCCHC14	Zinc finger CCHC-type containing 14
		ZHX2	Zinc finger and homeoboxes 2

Fig. S6. Minimal evidence for hypoxic regulation of known activators or repressors of HBV transcription. (a) RNA-Seq analysis of HepG2 cells treated with 1% O_2 for 16h, was interrogated for differential expression of known activators and repressors of HBV. Fold change was plotted against the FDR, to show the impact of hypoxia on the expression of these genes previously identified to promote or repress HBV transcription. Differential expression of known hypoxic response genes was plotted to confirm cellular response to low oxygen [18]. Dashed line indicates a fold change of + 2, which is the threshold for statistical significance. **(b)** TMT Proteomic data of Huh7 cells treated with 1% O_2 for 24h was interrogated for expression of activators and repressors from gene set as used in (a). Whole Cell Lysate values were used,

where hypoxic expression was normalised to the control sample, and converted to Log2FC. A panel of known hypoxic responsive genes was also checked to confirm the cellular response to low oxygen. Blue dotted line at Log2FC =1 indicated the threshold for statistical significance. Unless otherwise stated, all data presented as the mean ± SD derived from 3 independent experiments consisting of 3 replicates per condition. Statistical analysis was performed using a Mann-Whitney *U*-Test. (**c**) Table to show gene symbol and full gene names of previously reported activators, co-activators, repressors, and internal hypoxic control genes.

Supplementary references

[1] Hayer J, Jadeau F, Deleage G, Kay A, Zoulim F, Combet C. HBVdb: a knowledge database for Hepatitis B Virus. Nucleic Acids Res 2013;41:D566-570.

[2] McNaughton AL, D'Arienzo V, Ansari MA, Lumley SF, Littlejohn M, Revill P, et al. Insights From Deep Sequencing of the HBV Genome-Unique, Tiny, and Misunderstood. Gastroenterology 2019;156:384-399.

[3] Liu PJ, Harris JM, Marchi E, D'Arienzo V, Michler T, Wing PAC, et al. Hypoxic gene expression in chronic hepatitis B virus infected patients is not observed in state-of-the-art in vitro and mouse infection models. Sci Rep 2020;10:14101.

[4] Wing PA, Davenne T, Wettengel J, Lai AG, Zhuang X, Chakraborty A, et al. A dual role for SAMHD1 in regulating HBV cccDNA and RT-dependent particle genesis. Life Sci Alliance 2019;2.

[5] Ko C, Lee S, Windisch MP, Ryu WS. DDX3 DEAD-box RNA helicase is a host factor that restricts hepatitis B virus replication at the transcriptional level. J Virol 2014;88:13689-13698.

[6] Ko C, Chakraborty A, Chou W-M, Hasreiter J, Wettengel JM, Stadler D, et al. Hepatitis B virus genome recycling and de novo secondary infection events maintain stable cccDNA levels. Journal of Hepatology 2018.

[7] D'Arienzo V, Magri A, Harris JM, Wing PAC, Ko C, Rubio CO, et al. A PCR assay to quantify patterns of HBV transcription. J Gen Virol 2019.

[8] Michler T, Grosse S, Mockenhaupt S, Roder N, Stuckler F, Knapp B, et al. Blocking sense-strand activity improves potency, safety and specificity of anti-hepatitis B virus short hairpin RNA. EMBO Mol Med 2016;8:1082-1098.

[9] Guidotti LG, Matzke B, Schaller H, Chisari FV. High-level hepatitis B virus replication in transgenic mice. J Virol 1995;69:6158-6169.

[10] D'Arienzo V, Ferguson J, Giraud G, Chapus F, Harris JM, Wing PAC, et al. The CCCTC-binding factor CTCF represses hepatitis B virus enhancer I and regulates viral transcription. Cell Microbiol 2020:e13274.

[11] Perez-Riverol Y, Csordas A, Bai J, Bernal-Llinares M, Hewapathirana S, Kundu DJ, et al. The PRIDE database and related tools and resources in 2019: improving support for quantification data. Nucleic Acids Res 2019;47:D442-D450.

[12] Weekes MP, Tomasec P, Huttlin EL, Fielding CA, Nusinow D, Stanton RJ, et al. Quantitative temporal viromics: an approach to investigate host-pathogen interaction. Cell 2014;157:1460-1472.

[13] McAlister GC, Huttlin EL, Haas W, Ting L, Jedrychowski MP, Rogers JC, et al. Increasing the multiplexing capacity of TMTs using reporter ion isotopologues with isobaric masses. Anal Chem 2012;84:7469-7478.

[14] Ting L, Rad R, Gygi SP, Haas W. MS3 eliminates ratio distortion in isobaric multiplexed quantitative proteomics. Nat Methods 2011;8:937-940.

[15] Elias JE, Gygi SP. Target-decoy search strategy for increased confidence in large-scale protein identifications by mass spectrometry. Nat Methods 2007;4:207-214.

[16] Elias JE, Gygi SP. Target-decoy search strategy for mass spectrometry-based proteomics. Methods Mol Biol 2010;604:55-71.

[17] Huttlin EL, Jedrychowski MP, Elias JE, Goswami T, Rad R, Beausoleil SA, et al. A tissue-specific atlas of mouse protein phosphorylation and expression. Cell 2010;143:1174-1189.

[18] Smythies JA, Sun M, Masson N, Salama R, Simpson PD, Murray E, et al. Inherent DNA-binding specificities of the HIF-1alpha and HIF-2alpha transcription factors in chromatin. EMBO Rep 2019;20.
