# Supplementary material for: Hypoxia inducible factors regulate hepatitis B virus replication by activating the basal core promoter
Source: J Hepatol. 2021 Jul;75(1):64–73. doi: 10.1016/j.jhep.2020.12.034 (PMC8214165; doi:10.1016/j.jhep.2020.12.034)
Supplement: Multimedia component 2 [file mmc2.pdf]

JOURNAL
OF HEPATOLOGY

Journal of Hepatology

CTAT methods

Tables for a "Complete, Transparent, Accurate and Timely account" (CTAT) are now mandatory for all revised submissions. The aim is to enhance the reproducibility of methods.

- Only include the parts relevant to your study
- Refer to the CTAT in the main text as 'Supplementary CTAT Table'
- Do not add subheadings
- Add as many rows as needed to include all information
- Only include one item per row

If the CTAT form is not relevant to your study, please outline the reasons why:

1.1 Antibodies

Name	Citation	Supplier	Cat no.	Clone no.
Mouse anti-HIF-1α		BD Transduction Labs	610959	4073775
Mouse anti-HIF-2α		Novus	NB100-132	Q-4
Rabbit anti-HIF-1 β (ARNT1)		Novus	NB100-110	C-11
Mouse anti- β actin		Sigma	A5441	M2
Mouse anti-CAIX	Ioanna et al. 2015	A kind gift from Prof AL Harris		
Rabbit anti-HIF-1α ChIP	Mole, DR. et al. J Biol Chem 2009	A kind gift from Prof PJ Ratcliffe		
Rabbit anti-HIF-2α ChIP	Mole, DR. et al. J Biol Chem 2009	A kind gift from Prof PJ Ratcliffe		
Anti-RORA ChIP		Sigma	SAB4502650	3110554
Anti-HNF4α ChIP		Abcam	Ab181604	Gr3187190-12
Polyclonal goat anti-mouse immunoglobulins HRP		Dako	P0447	20062696
ECL anti-rabbit IgG, HRP linked whole antibody		GE Healthcare Life Sciences	NA934V	17041905
Alexa Flour 488 Mouse anti-BrdU		Biolgend	364105	B281715
Mouse Anti HBc		Santa Cruz	Sc23945	A2016

1.2 Cell lines

Name	Citation	Supplier	Cat no.	Passage no.	Authentication test method
HepG2-NTCP		Kind Gift from Prof Stephan Urban			Verified to be susceptible to HBV infection by ELISA
PHH		Thermofisher	**HU0028**		Verified to be susceptible to HBV infection by ELISA
HepAD38		Kind Gift from Prof Ullrike Protzer			Verified production of HBV
HepG2-pEpi		Kind Gift from Prof Ullrike Protzer			Verified presence of episomal HBV genome by qPCR

1.3 Organisms

Name	Citation	Supplier	Strain	Sex	Age	Overall n number
HBVtg mice	Michler,t. EMBO. 2016 Guidotti, LG JVirol 1995		HBV1.3.32	M/F	12-15 weeks	

1.4 Sequence based reagents

Name	Sequence	Supplier
siRNA HIF-1α	ND	Ambion; 4390824; s6539
siRNA HIF-2α (EPAS1)	ND	Ambion; 4390824; s4700
siRNA Scrambled (Negative Control)	ND	Ambion; 4390843
siRNA HIF-1b (ARNT1)	ND	Santa Cruz; sc-29733
Silencer Negative Control No.1 siRNA	ND	Ambion; AM4611
Mouse HIF-1b 1	ND	ThermoFisher; AM16708
Mouse HIF-1b 2	ND	ThermoFisher; AM16708
Negative control siRNA-1	ND	ThermoFisher; AM4404021

HBV-pgRNA TaqMan® Gene Expression Assay	ND	ThermoFisher; AIKAMSS
CAIX TaqMan® Gene Expression Assay	ND	ThermoFisher; Hs00154208_m1
VEGFA TaqMan® Gene Expression Assay	ND	ThermoFisher; Hs00900055_m1
B2M Control Mix	ND	Applied Biosystems; 4325797
PrP forward	TGCTGGGAAGTGCCATGAG	
PrP reverse	CGGTGCATGTTTTCACGATAGTA	
rcDNA forward	GTTGCCCGTTTGTCCTCTAATTC	
rcDNA reverse	GGAGGGATACATAGAGGTTCCTTGA	
cccDNA forward	GCCTATTGATTGGAAAGTATGT	
cccDNA reverse	AGCTGAGGCGGTATCTA	
4T PCR T1 forward	GGGGAACTAATGACTCTAGCTACC	
4T PCR T1 reverse	TTTAGGCCCATATTAGTGTTGACA	
4T PCR T2 forward	CAAGGTAGGAGCTGGAGCATTC	
4T PCR T2 reverse	GAGGCAGGAGGCGGATTTG	
4T PCR T3 forward	CTCCAGTTCAGGAACAGTAAACCC	
4T PCR T3 reverse	AGGAATCCTGATGTGATGTTCTCC	
4T PCR T4 forward	ACGGGGCGCACCTCTCTTTA	
4T PCR T4 reverse	GTGAAGCGAAGTGCACACGG	
~~HepBT1g forward~~	~~AGACCACCAAATGCCCCTATC~~	-
~~HepBT1g reverse~~	~~TTGAGATCTTCTGCGACGCG~~	-
HBV4F	TTTCACCTCTGCCTAATCATCTCT	
HBV4R	CTTTATAAGGGTCGATG CCATGC	
CAIX forward	TATCTGCACTCCTGCCCTCTG	
CAIX reverse	CACAGGGTGTCAGAGAGGGTGT	
VEGF forward	TTGCCTTGCTGCTCTACCTCCA	
VEGF reverse	GATGGCAGTAGCTGCGCTGATA	
NDRG1 forward	TTTGATGTCCAGGAGCAGGA	
NDRG1 reverse	ATGCCGATGTCATGGTAGGT	
B2M forward	CTACACTGAATTCACCCCCACTG	
B2M reverse	ACCTCCATGATGCTGCTTACATG	
B-Actin forward	CCAACCGCGAGAAGATGA	
B-Actin reverse	CCAGAGGCGTACAGGGATAG	
Mouse HIF-1b forward	CTCACGAAGGTCGTTCATCTGC	
Mouse HIF-1b reverse	CCACAAAGTGAGGTTCTCCTTCC	
Mouse VEGFA forward	CTGCTGTAACGATGAAGCCCTG	
Mouse VEGFA reverse	GCTGTAGGAAGCTCATCTCTCC	
Mouse PHD2 forward	GGCGAACGATTGAGGCTTCCTT	
Mouse PHD2 reverse	GCTGGTGACAGCAAAGAGAAGG	
Mouse B-Actin forward	CATTGCTGACAGGATGCAGAAGG	
Mouse B-Actin reverse	TGCTGGAAGGTGGACAGTGAGG	
ChIP HepB1 forward	GTCTGTGCCTTCTCATCTGCC	

ChIP HepB1 reverse	AGACCTTGGGCAATATTTGGTGG	
ChIP HepB3 forward	GGGGAGGAGATTAGGTTAAAGGTC	
ChIP HepB3 reverse	CAAGAGATGATTAGGCAGAGGTGA	
ChIP cccDNA forward	CCGTGTGCACTTCGCTTCA	Werle-Lapostolle B, Gastro. 2004
ChIP cccDNA reverse	GCACAGCTTGGAGGCTTGA	
ChIP CAIX forward	TCTCGTTTCCAATGCACGTACAGC	
ChIP CAIX reverse	AGTGACAGCAGCAGTTGCACAGT	
ChIP NDRG1 forward	TCCCTCCCAATCTCTCTCTTCTT	
ChIP NDRG1 reverse	CACCATCAGCACAGCAAACTAC	
ChIP BMAL1 forward	TTGGGCACAGCGATTGGT	
ChIP BMAL1 reverse	GTAAACAGGCACCTCCGTCC	
ChIP yGlobin forward	GCCTTGACCAATAGCCTTGACA	Groves, IJ. Oncogene 2016
ChIP yGlobin reverse	GAAATGACCCATGGCGTCTG	
ChIP APOB forward	GCATGTGAGGGTGAGGAAAT	Wang, SH. Gastro. 2012
ChIP APOB reverse	GAGTCCAGCTGCAGTGATGA	
ChIP HBV-RORE forward	TTACACACTCTATGGAAGGCGG	
ChIP HBV-RORE reverse	AAAGATTCTGCCCCATGCTGTA	

1.5 Biological samples

Description	Source	Identifier
Liver Biopsies	UPO; M Pirisi	

1.6 Deposited data

Name of repository	Identifier	Link
TMT Proteomics	XD020086	Data are available via ProteomeXchange with identifier PXD020086.

1.7 Software

Software name	Manufacturer	Version
Prism	GraphPad	8
LightCycler® 96	Roche	4
nSolver	nanoString	4
FlowJo	FlowJo LLC	V8.3

1.8 Other (e.g. drugs, proteins, vectors etc.)

Rabbit Serum	Sigma	20042349
FG-4592	MedChem Express	HY-13426
Bromodeoxyuridine	Sigma	B5002

1.9 Please provide the details of the corresponding methods author for the manuscript:

Prof. Jane A McKeating, Nuffield Department of Medicine, University of Oxford, Oxford, UK. Tel: (44) 1865 612894. Email: jane.mckeating@ndm.ox.ac.uk

2.0 Please confirm for randomised controlled trials all versions of the clinical protocol are included in the submission. These will be published online as supplementary information.

N/A
